# Impression of foliar-applied folic acid on coriander (*Coriandrum sativum* L.) to regulate aerial growth, biochemical activity, and essential oil profiling under drought stress

**DOI:** 10.3389/fpls.2022.1005710

**Published:** 2022-10-21

**Authors:** Muhammad Tajammal Khan, Shakil Ahmed, Rehana Sardar, Muhammad Shareef, Asim Abbasi, Muhammad Mohiuddin, Sezai Ercisli, Sajid Fiaz, Romina Alina Marc, Kotb Attia, Naeem Khan, Kiril S. Golokhvast

**Affiliations:** ^1^ Institute of Botany, University of the Punjab, Lahore, Pakistan; ^2^ Division of Science and Technology, Department of Botany, University of Education, Lahore, Pakistan; ^3^ Department of Botany, University of Narowal, Punjab, Pakistan; ^4^ Department of Environmental Sciences, Kohsar University, Murree, Pakistan; ^5^ Department of Horticulture, Faculty of Agriculture, Ataturk University, Erzurum, Turkey; ^6^ Department of Plant Breeding and Genetics, The University of Haripur, Haripur, Pakistan; ^7^ Food Engineering Department, Faculty of Food Science and Technology, University of Agricultural Sciences and Veterinary Medicine, Cluj-Napoca, Romania; ^8^ Department of Biochemistry, College of Science, King Saud University, Riyadh, Saudi Arabia; ^9^ Department of Agronomy, Institute of Food and Agricultural Sciences, University of Florida, Gainesville, FL, United States; ^10^ Siberian Federal Scientific Center of Agrobiotechnology, Russian Academy of Sciences (RAS), Krasnoobsk, Russia

**Keywords:** antioxidants, economic yield, field capacity, osmolytes, oil constituents, net photosynthesis, susceptibility index, principal component analysis

## Abstract

Drought is one of the major environmental limitations in the crop production sector that has a great impact on food security worldwide. Coriander (*Coriandrum sativum* L.) is an herbaceous angiosperm of culinary significance and highly susceptible to rootzone dryness. Elucidating the drought-induced physio-chemical changes and the foliar-applied folic acid (FA; vitamin B9)-mediated stress tolerance mechanism of coriander has been found as a research hotspot under the progressing water scarcity challenges for agriculture. The significance of folic acid in ameliorating biochemical activities for the improved vegetative growth and performance of coriander under the mild stress (MS75), severe stress (SS50), and unstressed (US100) conditions was examined in this study during two consecutive seasons. The results revealed that the plants treated with 50 mM FA showed the highest plant fresh biomass, leaf fresh biomass, and shoot fresh biomass from bolting stage to seed filling stage under mild drought stress. In addition, total soluble sugars, total flavonoids content, and chlorophyll content showed significant results by the foliar application of FA, while total phenolic content showed non-significant results under MS75 and SS50. It was found that 50 mM of FA upregulated the activity of catalase, superoxide dismutase, and ascorbate peroxidase enzymes in MS75 and SS50 plants compared with untreated FA plants. Thus, FA treatment improved the overall biological yield and economic yield regardless of water deficit conditions. FA-accompanied plants showed a decline in drought susceptibility index, while it improved the drought tolerance efficiency, indicating this variety to become stress tolerant. The optimum harvest index, essential oil (EO) percentage, and oil yield were found in MS75 followed by SS50 in FA-supplemented plants. The gas chromatography–mass spectrometry analysis revealed a higher abundance of linalool as the major chemical constituent of EO, followed by α-terpeniol, terpinene, and p-Cymene in FA-treated SS50 plants. FA can be chosen as a shotgun tactic to improve drought tolerance in coriander by delimiting the drastic changes due to drought stress.

## Introduction

The average global temperature is rising, and the environment is becoming more unpredictable with severe droughts occurring (Sheoran et al., 2022; Stocker et al., 2014). According to predictions, water scarcity would affect 1.5–1.7 billion people in South Asia by 2050 (Sheoran et al., 2021). Agriculture is the most vulnerable sector to this rapid climate change. Among many unprecedented challenges, drought is the most devastating abiotic stress caused by temperature dynamics, light intensity, and inadequate rainfall (Sheoran et al., 2022). According to FAO estimates, drought caused a direct loss of USD 29 billion to agriculture in developing countries between 2005 and 2015 (Canton, 2021). Each year, the global population and agriculture endure crucial agricultural output losses because of severe drought catastrophe. Despite this, its cumulative, subtle influence and multifaceted nature have a significant impact on plant morphological, physiological, biochemical, and molecular properties, with a negative impact on photosynthetic capacity (Bijalwan et al., 2022). Drought stress has a multidimensional impact on plant development, resulting in intricate and often spontaneous physiological and cellular responses leading to a substantial reduction in crop yield (Rehman et al., 2022).

Plants have acquired considerable physiological and biochemical adaptations in response to a variety of environmental stressors (Sharma et al., 2022). Drought can influence stomatal activity, which impacts CO_2_ absorption and, ultimately, the rate of photosynthesis and plant development. In response to a water scarcity, ion and water transport systems across membranes work to regulate fluctuations in guard cell turgor pressure and induce stomatal closure (Qi et al., 2018). During drought, endogenous ABA is rapidly produced, generating a cascade of physiological events, most notably the closure of stomata, which is controlled by a network of signaling molecules such as in *Arabidopsis* wherein drought stress activates 9-cis-epoxy carotenoid dioxygenase 3, which catalyzes a critical step in ABA synthesis (Endo et al., 2008; Behnam et al., 2013).

The overproduction of reactive oxygen species (ROS) causes decreased CO_2_ assimilation rates, while increased light absorption is another prevalent event under a drought environment, causing lipid peroxidation, DNA mutation, and cell damage (Kumar et al., 2019). These low levels, in turn, have an impact on the Calvin cycle processes, resulting in a decreased consumption of NADPH and ATP. As a response, the regeneration of electron acceptors declines, resulting in excessive ROS production (Chiappero et al., 2019; Rahimi et al., 2022). When ROS, such as hydrogen peroxide (H_2_O_2_), singlet oxygen (^1^O_2_), superoxide (O_2_), and the hydroxyl radical (OH), are present in minute amounts in plant tissues, they act as secondary messengers. However, when their production increases, the consequences of ROS become detrimental. The over-accumulation of ROS causes peptide chain fragmentation, deoxyribose oxidation, strand breakage, nucleotide elimination, and cell death (Cruz de Carvalho, 2008; Miller et al., 2010; Sharma et al., 2012). Plants have non-oxidative and oxidative enzymes, called scavenging enzymes, that assist in reducing this surplus. These include SOD, APX, CAT, glutathione reductase, and peroxidase (POX). These enzymes reduce the effects of ROS overabundance by oxidizing, reducing, and dismutating O_2_ to H_2_O_2_. However, during drought, the activity of these enzymes is changed and lowered, rendering them unable to carry out ROS-regulating tasks (Etesami and Maheshwari, 2018; Chiappero et al., 2019; Ilyas et al., 2021).

Recently, much emphasis has been made on the potential of using natural and safe compounds to boost plant development. FA (vitamin B9) is a well-known vitamin which is naturally synthesized in higher plants and influences plant growth and development (Ibrahim et al., 2021). It has a melting point of 482°F, and its molecular formula is C_19_H_19_N_7_O_6_. It is hydrophilic and slightly soluble in methanol, ethanol, and butanol-like organic solvents.

Folic acid is a collective term for pteroylglutamic acids and their oligoglutamic acid conjugates and natural water-soluble substance. It is N-acyl-amino acid that is a form of the water-soluble vitamin B9. Functionally, it relates to pterotic acid as a conjugate acid of folate.

Folic acid also has an auxinic function, which contributes to the growth, yield, and yield quality of many plant species. It is effective at capturing free radicals or active oxygen generated during photosynthesis and respiration processes (Hassan et al., 2016). FA-derived folates operate as glutamate residues, bridging the nitrogen and glucose metabolic processes (Stakhova et al., 2000). As a consequence, any interference with plant folate metabolism may result in a dramatic reduction in growth, development, and productivity by a considerable decrease in cell division and genome stability (Gorelova et al., 2017). Furthermore, it has been observed that the downregulation of genes involved in folate synthesis resulted in a considerable reduction or depletion of endogenous folates in many plant species in response to biotic and abiotic challenges (Higa et al., 2021). This response could indicate how essential the exogenous application of FA is for boosting plant tolerance to diverse stressors (Ibrahim et al., 2015; Kilic and Aca, 2016).

FA may be significant in regulating protein and nucleic acid synthesis, boosting cell division and expansion, stimulating the production of natural hormones and chlorophyll, and improving nutrition intake. These effects may explain why folic acid foliar spray enhances plant growth characteristics (Kilic and Aca, 2016). Increasing resilience to abiotic stress is also significant (Poudineh et al., 2015). A raise in the size of the entire vegetative development, as evidenced by an increase in plant biomass and dry matter accumulation, demonstrated the significance of folate in these activities (Khan et al., 2021). FA foliar spray to bean plants dramatically boosted the amount of total chlorophyll in Faba bean leaves. FA increased the amount of chlorophyll in leaves, which could be because it promoted the manufacture of glycine. Glycine is required for the formation of chlorophyll and porphyrins in chloroplast membranes (Stakhova et al., 2000).

In previous studies, it has been reported that the application of FA inhibited the negative impact of drought stress on plant development due to the production of proline under drought stress, which helps the plant to prevent turgor loss (Ibrahim et al., 2021). Moreover, proline is an amino acid that acts as osmo-protectant and molecular chaperons produced under the influence of the glutamate pathway during drought conditions, whereas, FA (pteroylglutamic acid) that naturally exhibits glutamic acid is directly involved in the formation of glutamate. This reaction is catalyzed by two enzymes (pyrroline-5-carboxylate synthetase and pyrroline-5-carboxylate reductase) in plants for the synthesis of proline. The production of proline in the cytosol dilutes the cell and prevents the dehydration of plant tissues (Sekhar et al., 2007; Shamsul et al., 2012), so this connection demonstrated a significant role of FA in biochemical and molecular processes and the development of plants (Esfandiari et al., 2012).

*Coriandrum sativum* L. is a medicinal annual plant in the Apeaceae family, which is useful in the pharmaceutical, food, cosmetic, and health sectors. It is of a herbaceous nature which, under abiotic stress, shows poor growth and develops a reduced leaf area (Drunasky and Struve, 2005). Under the progressing water shortage scenario across the globe, understanding the drought-induced physio-chemical changes and foliar-applied folic acid (FA; vitamin B9)-mediated stress tolerance mechanism of coriander has been found as a research hotspot which needed to be addressed on scientific grounds. Moreover, the literature was deprived of the revealing significance of foliage-applied folic acid in ameliorating the biochemical activities for the enhanced vegetative growth and performance of coriander under various intensities of drought stress. Therefore, a bi-seasonal experimentation was carried out using three irrigation levels with the following particular objectives: (i) to assess and quantify the physiological and biochemical performance and (ii) to evaluate the effectiveness of foliar FA in drought stress tolerance mechanism, physiological betterment, and metabolic enhancement of coriander for sorting out an optimal level of irrigation. This study would be an important addition to the knowledge of sustainable cultivation of Coriander with FA application under limited availability of water in the scenario of declining water resources across the globe.

## Materials and methods

### Description of the experimental site

The present study was carried out at the experimental area of the Department of Botany, University of Education Lahore Campus, Dera Ghazi Khan, Pakistan, during the crop seasons of 2019/2020 and 2020/2021 under natural conditions. This site lies at 30°06′ N (longitude) and 70°62′ E (latitude) at an elevation of 129 m above sea level. The climate of this vicinity is subtropical dry arid that is characterized by an annual average maximum temperature throughout summer of 42°C, while in winter the average minimum temperature is 4°C, with 104 mm average annual rainfall. The average temperature from November to April of the first and second season was 25 ± 3 and 23 ± 1°C, respectively. The total annual precipitation was 173 mm in the first season and 104 mm in the second season.

### Assessment of the physio-chemical properties of soil and water

The soil was clay loamy in texture, whereas the other characteristic properties are as follows: pH, 7.33; electrical conductivity, 2.13 ms/cm; field capacity, 37.27%; bulk density, 1.15 g/cm^-3^; organic matter, 0.78%; saturation, 32%; cations (K^+^, 0.01 meq/100 g; Na^+^, 0.29 meq/100 g; Ca^++^, 0.33 meq/100 g); anions (Cl^-^, 0.50 meq/100 g; CO_3_^-2^, 0 meq/100 g; HCO^-3^, 0.31 meq/100 g); and particle size distribution (sand, 37.9%; silt, 33.6%; and clay, 28.5%).

The characteristic properties of irrigated water were measured as follows: electrical conductivity (0.4 dS/m), cations (8.6 miliEq/L), anions (8.6 miliEq/L), sodium (1.29 miliEq/L), calcium (3.7 miliEq/L), magnesium (2.1 miliEq/L), chlorides (1.13 miliEq/L), sulphates (1.02 miliEq/L), total dissolved salts (256 ppm), carbonates (0.02 miliEq/L), bicarbonate (2.17 miliEq/L), sodium absorption ratio (0.98), and pH (7.11).

#### Calculation of the field capacity of soil

The field capacity (FC) of the two seasons was measured by the given formula in Equation 1. The calculated % of soil FC was 37.27 and 39.62% in 2019/20 (first season) and 2020/2021 (second season), respectively, in clay loamy-textured soil.


(1) FC (%) = (γw × V) / (γd × 1 x D) × 100

where γ_w_ is the weight of water, *V* is the volume of water, γ_d_ is the density of soil, and *D* is the root zone depth.

### Seed assortment and conditions for sowing

Certified seeds of coriander var Dilpazeer (lot no. FD-898602) were obtained from Ayub Agriculture Research Institute, Faisalabad, Pakistan. Sterilized seeds were sown on November 12, 2019 and November 18, 2020 at a depth of 1.5 cm with a density of 10–12 seeds per pot, and five plants per pot were maintained until seedling establishment. The pots were at all times irrigated to their respective field capacity with tap water after seedling establishment. Then, 50% FC was opted as benchmark for water replenishment in the experiment because coriander plants instantly wilt below this FC due to their drought-susceptible nature. To avoid the effects of the surroundings, the location of the pots was changed every week. Destructive sampling of plants was done at three different growth stages (vegetative stage after 45 days of sowing, bolting stage after 60 days of sowing, and seed filling stage after 75 days of sowing), and tissue and biochemical analyses were conducted before the seed filling stage, while the yield attributes were measured after harvesting. The pots were kept under natural conditions to match the almost field conditions. Weather forecast was monitored after every 4 h to make necessary arrangements before the account of a rainfall. Therefore, a rain-protector sheet (transparent) was used to prevent the effects of precipitation on the experiment.

### Procedure for water replenishment and FA application

After seedling establishment, the plants were subjected to drought stress based on maintenance of moisture content at field capacity (MCFC) by the replenishment method. The FC of soil was measured by saturating 7.2 kg potted test soil without plants with water and allowing the water to drench off completely for 24 h before weighing to take the initial weight (Iw). Then, this soil was dried at 105°C for 24 h and weighed for its final weight (Fw). Thereafter, the MCFC % was calculated by the given formula in Equation 2.


(2) MCFC % = (Iw−Fw/Fw)*100

The point of reference for the next irrigation was 50% of MCFC of full irrigation (US100). Therefore, setting 50% MCFC in US100 (control), 75 and 50% irrigation was applied to the rest of the plants, corresponding to 50% MCFC of full irrigation to generate water stress regimes (MS75—moderate level and SS50—intense level). Therefore, the depletion of water from field capacity was examined throughout the season by the usual soil sampling on a weekly basis through the gravimetric method (105°C and 24 h). In total, 7.2 kg of soil per pot contained 2.68 L of water in the first season, while it was 2.77 L water in the second season with respect to their soil moisture content at FC (%) that was used as full irrigation or control (US100). Thus, 50% of soil moisture at FC of the first and second seasons was 1.34 and 1.39 L of water, respectively. Hence, setting 50% FC of soil in pots of control plants (US100), 50% (0.67 and 0.70 L) and 75% (1.005 and 1.042 L) irrigation was applied to rest of the plants to generate water deficit regimes SS50 and MS75 in the first season and the second season. The same protocol was followed in maintaining the irrigation throughout the experiment until harvest. Drought stress was applied on December 9, 2019 and December 15, 2020 after seedling establishment. Foliar application of FA was initiated from the 10th day (December 19, 2019 and December 25, 2020) of applying drought stress and continued at a regular interval of 10 days for three times (6 ml for each pot).

### Pilot design and growth conditions

In this experiment, the influence of irrigation regimes including drought stress (US100, MS75, and SS50) on the growth, physiological, biochemical, metabolic, and essential oil characteristics of coriander and its adaptive response to drought stress under foliar-applied FA were examined. In our previous study, 50 mM FA showed a positive impact on coriander growth attributes in the pot experiment (Khan et al., 2021). By opting for 50 mM FA, the current experiment was designed, consisting of six treatments (TRT1 = US100, TRT2 = US100 + FA, TRT3 = MS75, TRT4 = MS75 + FA, TRT5 = SS50, and TRT6 = SS50 + FA) with four replicates. To evaluate the performance of three irrigation regimes and two levels of FA (control and 0 and 50 mM), the distance of each pot was 10 in. from each other in rows and 15 in. in columns. The size of the pot was 502 in.^3^, and all pots were replicated under natural conditions. Each pot was supplemented with 0.5 g urea and 0.3 g Di ammonium phosphate before sowing and first irrigation. Furthermore, it was evident from literature that FA is extremely susceptible to photolysis in aqueous solutions exposed to sunshine, so we chose to treat the plants in the evening when light is poor (Stakhova et al., 2000).

### Assessment of coriander growth at different stages

The growth attributes of *C. sativum* such as plant fresh biomass (PFB), plant dry biomass (PDB), leaf fresh biomass (LFB), leaf dry biomass (LDB), shoot fresh biomass (SFB), and shoot dry biomass (SDB) were studied at the vegetative, bolting, and seed filling stages through destructive plant sampling, weighing fresh samples, and oven drying.

### Estimation of gaseous exchange and water scenario

Portable Photosynthesis System of CID Bio-Science Inc. (model: CI 340, USA) was used to measure the photosynthetic rate (*P*_N_), transpiration rate (*E*), and stomatal conductance (*C*) from two totally expanded leaves of the upper canopy (Mitchell, 1992). These measurements were obtained from 9:30 a.m. to 11:30 a.m. The quantum flux of the photosynthesis system was customized according to a sunny situation so that sunlight was used as the light source.

Two completely expanded young leaves were used to measure the leaf water potential (**Ψ**_w_) and the relative water content (RWC) from 10:30 a.m. to 12:00 p.m. Water potential was measured by using a pressure chamber (model: 610, PMS INSTRUMENT Co. USA). Leaf discs (5 mm in diameter) were taken—having a fresh weight (W_f_) of 0.5 g—from each treatment and put into test tubes containing distilled H_2_O for 4 h at 4°C to determine the RWC. The saturated leaves were weighed (Wt) and then dried for 72 h at 65°C for the dry weight. RWC was estimated by using the formula given by González and González-Vilar (2001):


(3) RWC (%) = (Wf−Wd)/(Wt−Wd)*100

### Quantification of antioxidant enzyme activity

One gram of plant leaf tissue was homogenized in 1 ml of 0.1 M sodium phosphate buffer (pH 6) at 4°C and centrifuged at 12,000 rpm for 15 min, and the supernatants were stored at −80°C until use for the assay of peroxidase (POD) and catalase (CAT) activities. The POD activity was evaluated by following the (Hammerschmidt et al., 1982) protocol using guaiacol as substrate and considering that an increase in absorbance at 470 nm min^-1^ equals the POD activity (Liang et al., 2011). The CAT activity was assayed by calculating the decrease in H_2_O_2_ at an optical density of 240 nm considering that one unit of activity is equivalent to the CAT quantity that breaks down 1 μmol of H_2_O_2_ in 1 min (Worthington, 1988; Weisany et al., 2012). The activity of Superoxide Dismutase (SOD) enzyme was assayed as described by McCord and Fridovich (1969) and Paoletti et al. (1986) and calculated as Zhang et al. (2016). The activity is indicated as the number of units. One unit is defined as the amount of SOD that inhibits 50% of NADH oxidation. The method of Nakano and Asada (1987) was used to measure the activity of APX due to ascorbate oxidation based on H_2_O_2_. This decomposition of ascorbate determined the decrease in absorbance at 290 nm. Moreover, 1 ml of the mixture (potassium phosphate = 50 mM, ascorbate = 0.5 mM, and EDTA = 0.1 mM) reacted with H_2_O_2_ (0.1 mM) for the oxidation of ascorbate. The APX activity was evaluated as the quantity of enzymes responsible for the production of oxidized ascorbate at the rate of 1 µmol/min.

### Estimation of chlorophyll a and b

Chlorophyll a and b content (mg/g) was calculated using the method of Arnon (1949) before the seed filling stage. In total, 1 g of finely diced fresh leaves was crushed with a solution of one part of 0.1 normal (N) ammonium hydroxide solution to nine parts of acetone (v/v, volume to volume). Then, it was centrifuged (model; HC-1014, ZONKIA, China) for 5 min at 5,000–10,000 rpm, and the supernatant was diluted to a quantity that produces an absorbance value of between 0.2 and 0.8 at wavelengths of 663 and 645 nm. The absorbance of each solution is measured compared with a blank solvent, and the content of chlorophyll a and b was determined through a spectrophotometer (model; UV-2505, Lambomed Spectro, England).


(4) Chl a (µg/ml) = 12.7 (A663) − 2.69 (A645)

(5) Chl b (μg/ml) = 22.9 (A645) − 4.68 (A663)

### Assessment of osmolytes (proline and TSS)

Proline (PRO) content (µM/g.f.wt.) estimation was carried out using the method described by Bates et al. (1973). In total, 250 mg of leaves was blended in 5 ml of 3% sulpho salicylic acid and centrifuged (model; HC-1014, ZONKIA, China) at 10,000 rpm for 10 min. Moreover, 2 ml of the supernatant was combined with 2 ml of glacial acetic acid and 2 ml of acid ninhydrin reagent and then incubated at 100°C for 1 h. The reaction was stopped by immersing the tubes in an ice bucket, and the reaction mixture was extracted with 5 ml of toluene and thoroughly mixed with a vortex (model; Vortex Mixer XH-D, Wincom Co., China) for 15–20 s. The toluene from chromophore was extracted from the aqueous medium and kept at room temperature, and the absorbance at 520 nm was measured using toluene as a blank. The PRO content was calculated from a standard curve (as μmol PRO g Fw^−1^) using L-PRO as standard.

TSS was measured by following the method of Jayaraman and Jayaraman (1981). In total, 0.5 g of fresh leaves was taken and ground to liquid nitrogen along with 5 ml of ethanol (95%) to discharge out sugar, and then 5 ml of ethanol (75%) was added; the mixture was centrifuged at 4,000 rpm for 15 min. This solution was kept in the refrigerator at below 4°C for 1 week. The fresh anthrone reagent was prepared by adding 150 mg of anthrone into 100 ml of H_2_SO_4_ (72%). Then, 0.1 ml of stored ethanolic extract was taken and mixed with anthrone reagent of 3 ml, and this was put into the water bath at 95°C. A UV spectrophotometer was operated to measure the absorbance at 625 nm.

### Measurement of TPC

Dried leaves (10 g) from each treatment were obtained for the preparation of leaf extract. The samples were added with 75 ml of ethanol (95% v/v) for 10 min at 40°C. This extraction method was carried out three times. The extract was heated to evaporate the solvent at 40°C. The dried extract was used for further analysis. About 20–50 mg of the dried extract was added in 5 ml methanol and sonicated for 45 min (Ultra-sonicated bath, Branson 2510) at 40°C trailed by centrifugation for 10 min at 1,200 rpm. A clear supernatant was obtained and stored it amber glass bottle for further analysis.

Singleton and Rossi in 1965 measured the TPC of leaf extract by using the Folin–Ciocalteu reagent, with some modifications. Sample and standard curve readings were measured through a spectrophotometer (model; UV-2505, LambomedSpectro, England) at 765 nm against the blank (reagent). A given sample (0.2 ml) was mixed well with the Folin–Ciocalteu reagent (0.2 ml) and added with water (0.6 ml) by 1:1. Now, after passing for 5–8 min, a saturated solution of NaCO_3_ (8% w/v) was obtained. Then, 1 ml of saturated solution was taken and supplemented into the mixture. Now, distilled water was added to adjust the volume up to 3 ml. This reaction combination was placed in the dark for 35 min. Afterwards, the solution was centrifuged for 8 min at 4,000 rpm. The absorbance peaks of the supernatant (blue color) were observed at 765 nm. The TPC was measured as GA. Eq/g d. wt. (gallic acid equivalent) with respect to the standard calibration curve of GA (0 to 100 mg/ml).

### Quantification of TFC

The TFC was measured through the aluminum colorimetric method (Meda et al., 2005). For the determination of TFC, the standard calibration curve of quercetin (C_15_H_10_O_7_) was used. A stock solution of quercetin was formed by adding 5 mg C_15_H_10_O_7_ in 1 ml methanol (CH_4_OH); then, a standard concentration of C_15_H_10_O_7_ (0.25 to 1 mg/ml) was prepared by serial dilutions using CH_4_OH. Furthermore, 1 ml of each concentration was taken in a test tube and poured with distilled water (4 ml). Now, 5% of NaNO_2_ (0.3 ml) and 10% of AlCl_3_ (0.3_ ml_) water were added into the test tubes after 5 min. Then, 1 M of NaOH (2 ml) was added after 5 min, and the volume was increased to 10 ml by adding distilled water. After stirring, the mixture was placed for 60 min at 25°C. A UV spectrophotometer was used to calculate the absorbance at 510 nm against blank. The amount of TFC in the test samples was estimated from the standard calibration curve and expressed as milligram quercetin equivalent (QE)/g of dried weight.

### Measurements of agronomic parameters

The yield attributes were estimated as BY = seed + dry biomass, EY = seed yield, and HI = (EY/BY) × 100 (Donald and Hamblin, 1976).

### Assessment of DSI

DSI was estimated by the given equation with some modifications (Fischer and Maurer, 1978):


(6) DSI = (1−EYs/EYi)/A

whereas EY_s_ = economic yield under drought stress, EY_i_ = economic yield under full irrigation, and *A* = average economic yield of all treatment under drought stress/average economic yield of all treatments under full irrigation.

### Magnitude of DTE

The formula of Fischer and Maurer (1978) was employed for the estimation of DTE as given below:


(7) $$\text{DTE}=\frac{\text{EY under drought}}{\text{EY under full irrigation }\left(\text{US}100\right)\;}\times 100$$

### Isolation and determination of EO

One hundred grams of air-dried coriander seeds of the first and second seasons were used for hydro-distillation (2.5 h) for the extraction of oil by using Neo type Clevenger apparatus (Guenther, 1972). EO was expressed in percentage. EO% and OY (oil yield L/ha) were determined through the given formulas:


(8) $$\text{EO\% }=\;V_{EO}\;\times \;100/\;W_{EO}$$

(9) OY = EO% × EY/ 100

where *V*_EO_ = volume of essential oil extracted, *W*_EO =_ weight of seed used for essential oil extraction, and EY = economic yield (kg/ha).

The content of essential oil was measured through gas chromatography and mass spectrometry (GC–MS).

### Elucidation of the GC–MS of EO

The chemical component of EO was determined through gas chromatography together with mass spectrometry accomplished by using TRACE 1300 GC–MS (Single Quadrupole MS) of Thermo Scientific (USA) along with an auto-sampler (AI 1310). The column was of a non-polar phase, 5% polysilphenylene siloxane, and has the following dimensions: 30 m × 0.25 mm (*i*.*e*., film depth of 0.25 μm); TR-5MS (same as D-5MS) was used. The temperature program was adjusted initially at 60°C with increases of 4°C/min to 250°C/min (final temperature). The temperature of the injector was adjusted to 270°C, and the interface temperature was 280°C. The spectral scan range of the detector was 55–600 amu. The carrier gas was helium (He), with movement of 1.4 ml/min. The ionization voltage was 70 eV. Compounds were detected and identified by comparing their spectra to those in the Wiley NBS75K MS library and then verified by comparison.

### Statistical analysis/retrospective

This study laid out in a randomized complete block factorial design and the recorded data were analyzed statistically by using STATISTICS 8.1 and MS Excel 2016. The values were reported as means of four replications with standard error of the means (SE). By using analysis of variance (ANOVA), the raw data of the replicates of each treatment was analyzed at 5% probability to see the independent as well as combined effect of treatments. Both treatments (irrigation regimes and folic acid) were equally important—that is why factorial design was used. In ANOVA, the independent as well as correlative statistical means of each treatment were sorted for better comparison in least significant difference (LSD) test and vice versa. Therefore, LSD based on ANOVA test was applied at probability *P <*0.05 to treatment means for ranking and comparison. Bars exhibited with different letters indicate a significant difference between treatments by LSD (*p* ≤ 0.05). Principal component analysis (PCA) was performed using Origin 8.1 Pro software.

## Results

### Assessment of aerial growth parameters

**Figure 1:**
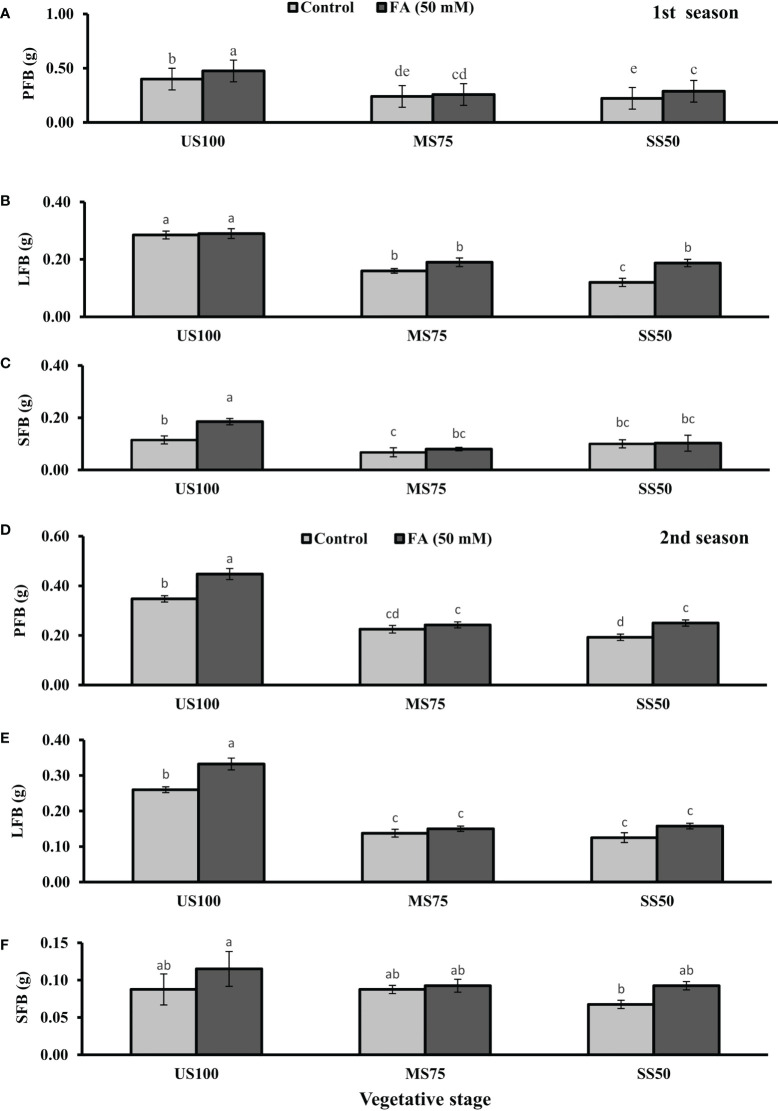
Effect of foliar application of folic acid (50 mM) at the vegetative stage on **(A, D)** plant fresh biomass/plant, **(B, E)** leaf fresh biomass/plant, and **(C, F)** shoot fresh biomass in the first and second seasons. The graph values are the mean ± SE of four replicates. The bars exhibited with different letters indicate a significant difference between samples by least significant difference (*p* ≤ 0.05).

**Figure 2:**
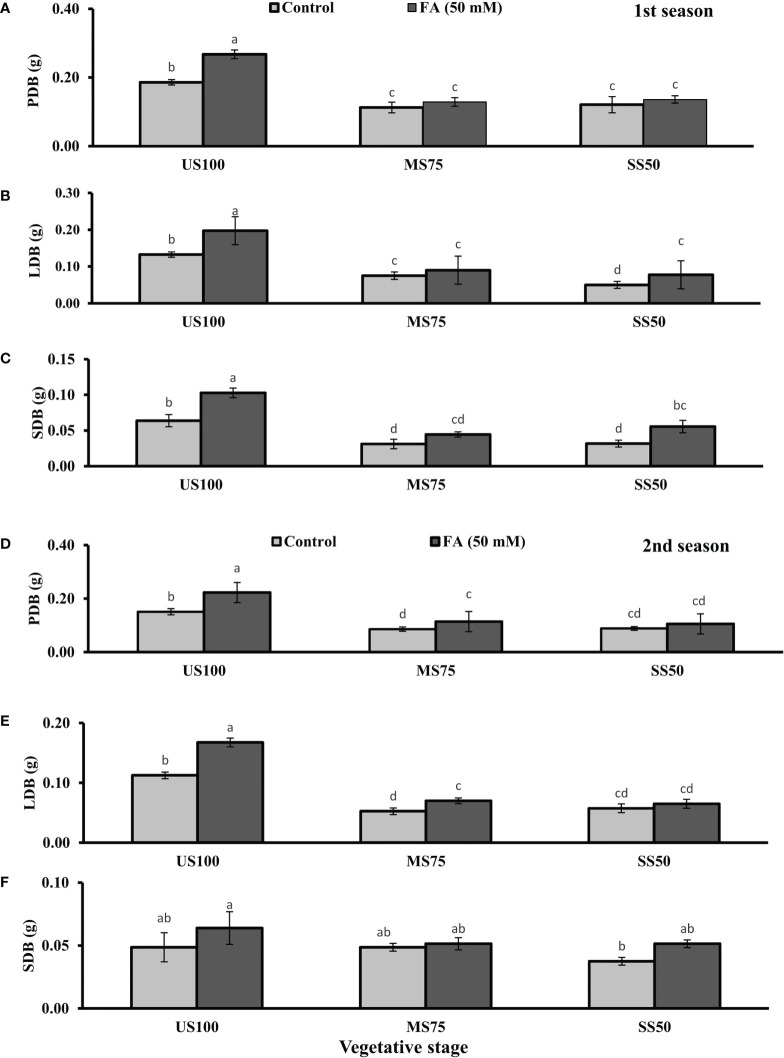
Effect of foliar application of folic acid (50 mM) at the vegetative stage on **(A, D)** plant dry biomass/plant, **(B, E)** leaf dry biomass/plant, and **(C, F)** shoot dry biomass/plant in the first and second seasons. The graph values are the mean ± SE of four replicates. The bars exhibited with different letters indicate a significant difference between samples by least significant difference (*p* ≤ 0.05).

The growth parameters like PFB, LFB, SFB, PDB, LDB, and SDB were measured at the vegetative, bolting, and seed filling stages. The ANOVA showed significant results of two levels of drought as shown in **Figures 1**, **2** followed by LSD. Drought stress decreased the PFB by 40% (0.24 g) and 45% (0.22 g) and PDB by 43% (0.11 g) and 37% (0.12 g) of coriander L. in MS75 and SS50 plants with respect to US100 plants (0.40/0.19 g) in the first season at the vegetative stage. Similarly, in the second season, drought stress significantly decreased the PFB by 34% (0.23 g) and 46% (0.19 g) and PDB by 40% each (0.09 g) in MS75 and SS50 compared with control plants (0.35 and 0.15 g) at the vegetative stage.

The foliar application of 50 mM FA significantly increased the fresh biomass of plant (9% and 32%) in the first season (4 and 32%) and second season under both drought regimes (MS75 and SS50) as well as in US100 (20 and 29%) compared with untreated FA plants as presented in **Figures 1A, D**. The PDB of coriander significantly increased in US100 (43 and 47%) and MS75 (19 and 23%) on account of foliar spray of FA in the first and second seasons corresponding to untreated FA plants as presented in **Figures 2A, D**. The FA-treated plants made no change in SS50 in both seasons compared with control plants. These findings proposed that FA has an interactive effect that enhanced the PFB of coriander remarkably in SS50 plants followed by MS75 plants, but no significant affect was found in the PDB of SS50 plants at the vegetative stage.

Furthermore, LDB significantly decreased by 39 and 55% in MS75 plants and 62 and 46% in SS50 plants in both seasons, respectively (**Figures 2B, E**). LFB significantly increased in the first season by 19% (0.19 g) in MS75 and 59% (0.19 g) in SS50 plants in drought compared with the control plants (0.16 and 0.12 g) as presented in **Figure 1B**. Meanwhile, in the second season, the increase was 8% (0.15 g) in MS75 plants and 23% (0.16 g) in SS50 plants set against untreated FA plants (0.14 and 0.13g). The LDB significantly increased in all levels of drought stress, including in the control. The maximum increase was found in SS50 plants (60%) followed by US100 plants (54%) in the first season compared with untreated FA control (**Figure 2B**). In the second season, the maximum increase was found in US100 (55%) trailed by MS75 (40%) in comparison with the control at the vegetative stage as shown in **Figure 1E**.

The SFB showed significant effects in results compared with the control plants (0.12 and 0.09 g) in the first and second seasons at the vegetative stage. Under drought stress, the vegetative stage plants of MS75 and SS50 showed that SFB decreased to 42% (0.07 g) and 17% (0.10g) in the first season, while in the second season this decrease was 23% as shown by SS50 only (**Figures 1C, F**). After the foliar spray of FA, SFB showed a significant increase in US100 plants (59%) and MS75 plants (15%), while there was no change at SS50 with respect to untreated FA plants in the first season (**Figure 1C**). In the second season, non-significant results were observed in US100 (34%) and SS50 plants (29%), and no changes were found in MS75 compared with control plants as shown in **Figure 1F**. The statistical analysis showed that the main effect of both drought regimes was non-significant in the case of SDB at the vegetative stage, while the main effect of FA and the interaction between drought levels and FA were significantly different from each other in both seasons as presented in **Figures 2C, F**.

**Figure 3:**
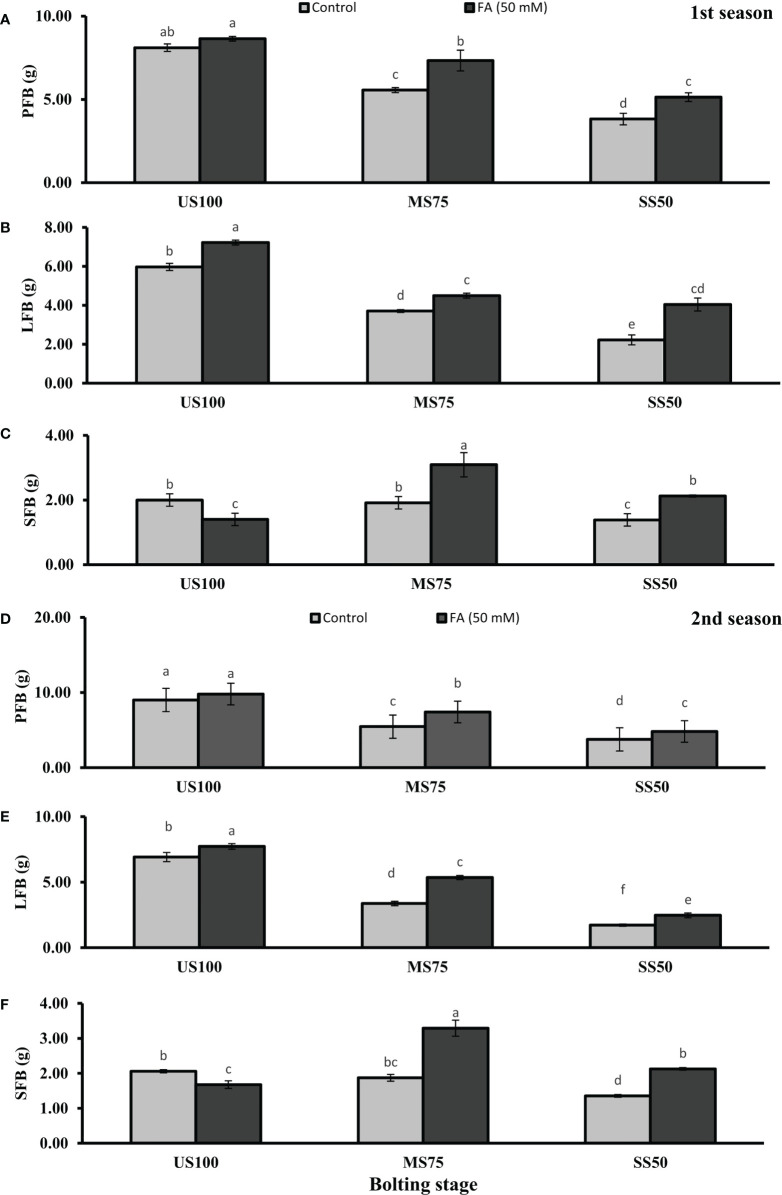
Effect of foliar application of folic acid (50mM) at bolting stage on **(A, D)** plant fresh biomass/plant, **(B, E)** leaf fresh biomass/plant, and **(C, F)** shoot fresh biomass in the first and second seasons. The graph values are the mean ± SE of four replicates. The bars exhibited with different letters indicate a significant difference between samples by least significant difference (*p* ≤ 0.05).

**Figure 4:**
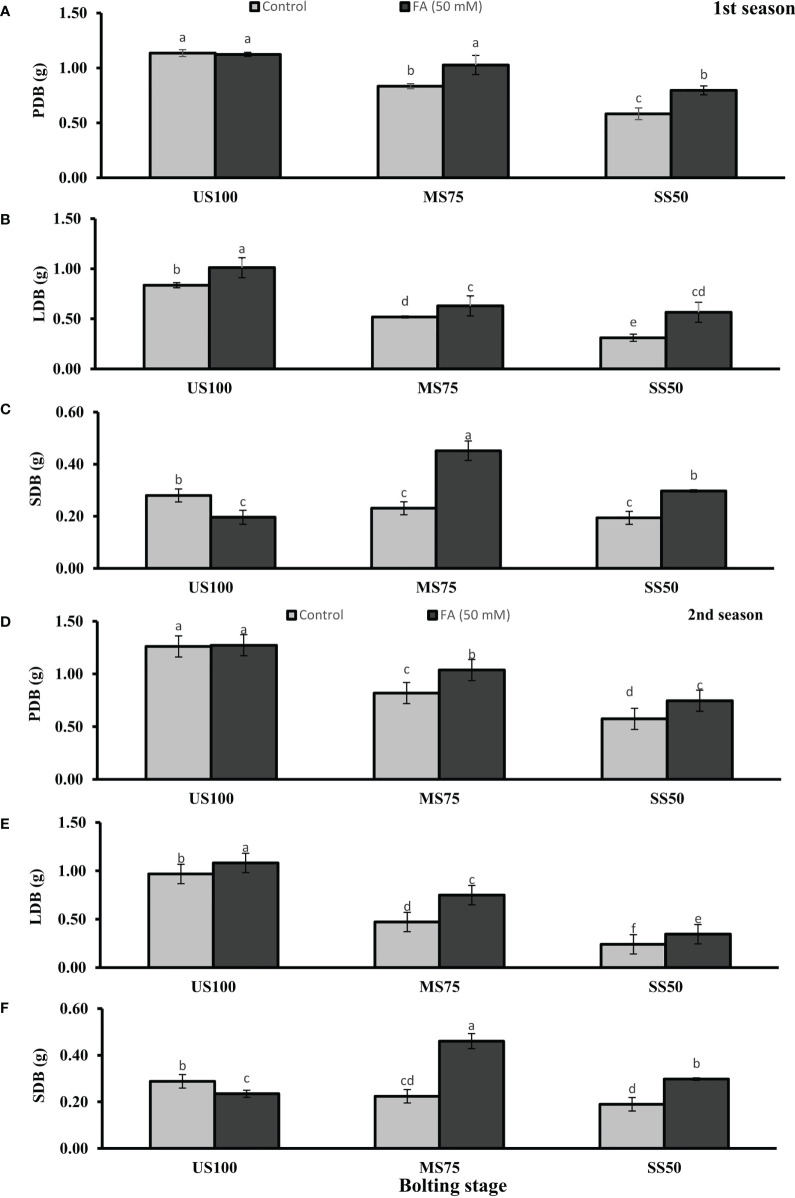
Effect of foliar application of folic acid (50 mM) at bolting stage on **(A, D)** plant fresh biomass/plant, **(B, E)** leaf fresh biomass/plant, **(C, F)** and shoot fresh biomass in the first and second seasons. The graph values are the mean ± SE of four replicates. The bars exhibited with different letters indicate a significant difference between samples by least significant difference (*p* ≤ 0.05).

Meanwhile, **Figures 3A**, **4A** showed a decrease of PFB by 32% (5.56 g) and 53% (3.82 g) and of PDB by 27% (0.83 g) and 54% (0.58 g) of coriander L. in MS75 and SS50 compared with US100 plants (8.11g/1.14g) in the first season at bolting stage. Similarly, in the second season drought stress significantly decrease the PFB by 39% (5.56g) and 58% (3.77g) and PDB by 35% (0.82g) and 55% (0.57) in MS75 and SS50 compared with fresh (9.01g) and dry biomass (1.26g) of control (US00) plants (**Figures 3D**, **4D**). The foliar application of 50 mM FA significantly increased the fresh biomass of plant in the first season (32 and 35%) and in the second season (36 and 28%) in both drought levels (MS75 and US50) as well as in US100 (6 and 9%) compared with untreated FA drought-stressed and unstressed plants as presented in **Figures 3A, D**. The PDB of coriander significantly increased in MS75 plants (24 and 27%) and SS50 plants (38 and 32%) on account of foliar spray of FA in the first and second seasons in comparison with untreated FA plants as presented in **Figures 4A, D**. These findings proposed that FA enhanced the PFB of coriander remarkably in MS75, and ANOVA indicates a significant effect of FA on PDB in SS50 plants at the bolting stage.

Water deficit conditions lessened the LFB in MS75 plants (38 and 51%) and SS50 plants (63 and 75%) in two seasons (**Figures 3B, E**) at the bolting stage. Furthermore, LDB was significantly reduced (38 and 52%) in MS75 and SS50 (63 and 75%) in both seasons, respectively (**Figures 4B, E**). LFB significantly increased on the application of FA in the first season 21% (4.50 g) in MS75 and 81% (4.04 g) in SS50 under drought regimes compared with control plants (3.71 and 2.23 g) as presented in **Figure 3B** for the bolting stage, whereas in the second season the increment was 59% (5.35 g) in MS75 plants and 44% (2.47 g) in SS50 plants w.r.t untreated plants (3.37 and 1.72 g) as presented in **Figure 3E**. The LDB significantly increased in all regimes as in MS75 plants (21 and 60%), SS50 plants (84 and 46%), and control plants (20 and 11%). Overall, the maximum improvement was found in SS50 followed by MS75 in the first season and *vice versa* in the second season in the case of LFB as well as LDB compared with untreated FA plants at the bolting stage.

The SFB and SDB showed significant results at the bolting stage compared with the control in the first (2 and 0.28 g) and the second (2.06 and 0.29 g) seasons. The plants of MS75 and SS50 decreased the SFB by 4% (1.92 g) and 31% (1.39 g) in the first season, while in the second season this decline in SFB was 9% (1.87 g) and 35% (1.35 g) as shown in **Figures 3C, F**. After the foliar spray of FA, the SFB showed significant results in MS75 plants (61%) and SS50 plants (53%) with respect to untreated FA plants in the first season, whereas in the second season, better results were observed by improving the SFB in MS75 plants (76%) and SS50 plants (58%) related to control plants at the bolting stage. The statistical analysis showed that the main effect of both drought levels was significant in the case of SDB. Statistically, the main effect of drought regimes and foliar application of FA and the interaction between them were significantly different in both seasons as presented in **Figures 4C, F**. FA treatment enhanced the SDB in MS75 plants (almost onefold and more than onefold) and SS50 plants (58% each) in both seasons associated to untreated FA plants at the bolting stage. In US100 plants treated with FA, it reduced the SFB and SDB in consecutive seasons with respect to untreated plants. These results showed that FA potentially increased the SFB and SDB at the bolting stage under drought stress with a maximum increase in MS75 followed by SS50.

**Figure 5:**
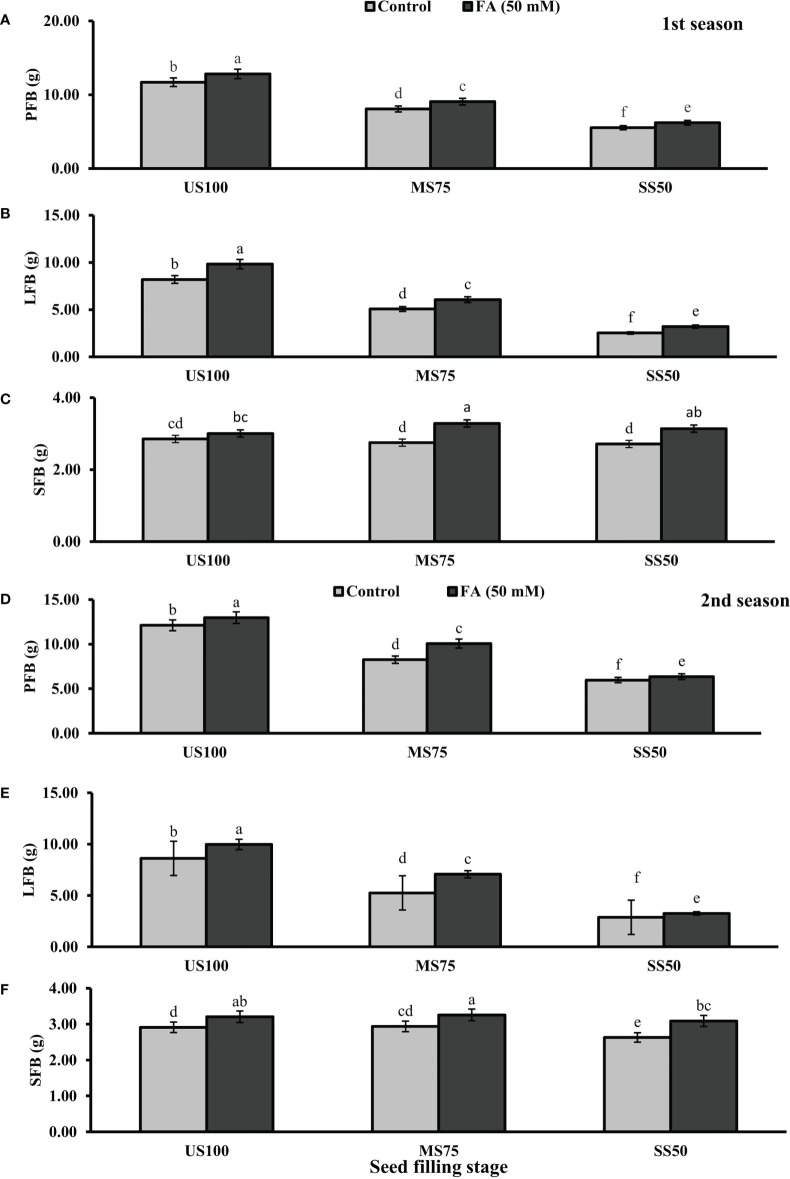
Effect of foliar application of folic acid (50 mM) at seed filling stage on **(A, D)** plant fresh biomass/plant, **(B, E)** leaf fresh biomass/plant, and **(C, F)** shoot fresh biomass in the first and second seasons. The graph values are the mean ± SE of four replicates. The bars exhibited with different letters indicate a significant difference between samples by least significant difference (*p* ≤ 0.05).

**Figure 6:**
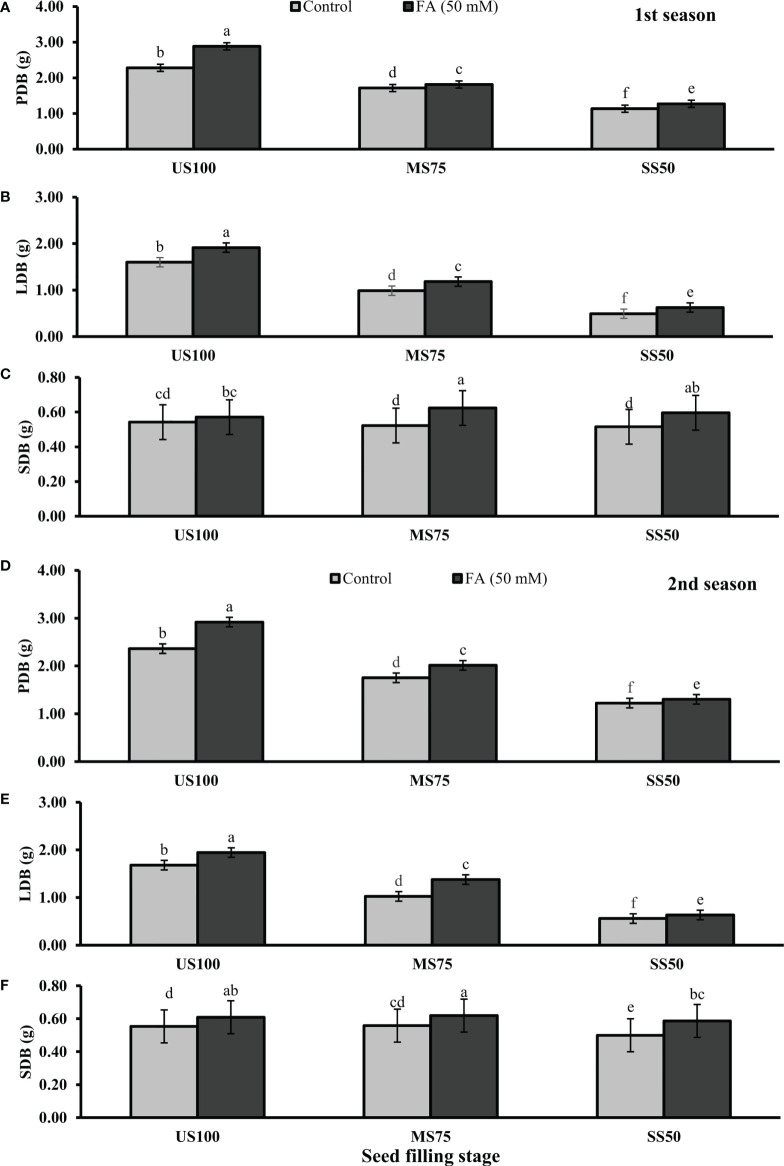
Effect of foliar application of folic acid (50 mM) at seed filling stage on **(A, D)** plant fresh biomass/plant, **(B, E)** leaf fresh biomass/plant, and **(C, F)** shoot fresh biomass in the first and second seasons. The graph values are the mean ± SE of four replicates. The bars exhibited with different letters indicate a significant difference between samples by least significant difference (*p* ≤ 0.05).

Statistically significant effects of drought regimes were obtained at the seed filling stage as shown in **Figures 5**, **6**. Drought stress decreased the PFB by 27% (8.07 g) and 53% (5.53 g) and PDB by 25% (1.71 g) and 50% (1.13 g) of coriander L. in MS75 and SS50 with respect to US100 plants (11.70/2.28 g) in the first season. Similarly, in the second season, drought stress significantly decreased the PFB by 32% (8.25 g) and 51% (5.97 g) and PDB by 26% (1.75 g) and 48% (1.22 g) in MS75 and SS50 compared with the control plants at the seed filling stage. Foliar application of 50 mM FA increased the PFB in the first season (12 and 12%) and in the second season (12 and 7%) in both drought regimes (MS75 and SS50) as well as in US100 (10 and 7%) compared with the untreated FA and fully irrigated plants as presented in **Figures 5A, D** on the set of seed filling stage. The PDB of coriander significantly increased in US100 (27 and 24%), MS75 (6 and 15%), and SS50 plants (12 and 7%) after FA supplementation in both seasons in contrast to untreated FA plants as presented in **Figures 6A, D**. These findings proposed that, at the seed filling stage, FA and drought regimes have a strong interaction that enhanced the PFB of coriander remarkably in MS75 followed by SS50, but the maximum significant increment in PDB was found in US100.

The results showed that drought stress significantly decreased the LFB in MS75 plants (38 and 39%) and SS50 plants (69 and 67%) at the seed filling stage. LFB significantly increased on the usage of FA in the first season by 20% (0.06 g) in MS75 and 27% (3.21 g) in SS50 related to US100 plants as presented in **Figure 5B**, whereas in the second season the increase was 35% (7.07 g) in MS75 and 14% (3.26 g) in SS50 with respect to the control plants (5.25 and 2.87g) during the seed filling stage. While under full irrigation (US100 plants), the increment in LFB was 18% in the first season and 16% in the second season in FA-treated plants. The application of FA significantly increased the LDB in both regimes of drought stress including in the control. The maximum increase was found in SS50 plants (29%) followed by US100 (20%) in the first season compared with untreated FA plants. In the second season, the maximum increase was found in MS75 plants (35%) trailed by US100 (16%) in comparison with the control plants as shown by **Figures 6B, E**.

The SFB showed a significant effect in the results compared with US100 plants (0.12 and 0.09 g) in the first and second seasons at the seed filling stage. MS75 plants showed non-significant results, while SS50 plants decreased the SFB to 5% (2.71 g) and 10% (2.63 g) in both seasons, respectively, compared with the fully irrigated plants (**Figures 5C, E**). Plants treated with FA showed a significant increase in SFB to 6, 19, and 16% in US100, MS75, and SS50 with respect to their untreated FA plants in the first season (**Figure 5C**). In the second season, a similar trend was observed in US100 (10%), MS75 (11%), and SS50 plants (18%) compared with the control as shown in **Figure 5F**. The statistical analysis showed that the main effect of both drought levels was non-significant in the case of SDB, while those plants treated with FA showed that the main effect of treatments and the interaction between drought regimes were significantly different from each other in both seasons as presented in **Figures 6C, F**.

### Effect of different treatments on gaseous exchange parameters and water status

The gaseous exchange parameters like photosynthetic active radiation (PAR), transpiration rate (E), stomatal conductance (S), internal carbon dioxide (Int. CO_2_), and net photosynthetic rate (Pn) were measured at three different growth stages to evaluate the effect of FA (50 mM) under two regimes of drought (MS75 and SS50) and in the control plants. The maximum value of PAR at the vegetative stage was found in TRT2, and the minimum value was observed in TRT5 in the first season. Meanwhile, in the second season, the maximum value of PAR was in TRT1, and the minimum value was observed in TRT5 at the vegetative stage. At the bolting stage, the TRT4 plants showed optimum results followed by TRT6 plants when treated with FA under stress regimes MS75 and SS50 (**Table 1**) in both seasons. The values of PAR are drastically decreased at the seed filling stage. Significantly different values were found in each treatment in both seasons as shown in **Table 1**. The range of PAR was 456.55 and 407.25 µm/m^2^/s (TRT2) to 379.75 and 329.25 µm/m^2^/s (TRT4) in the first and second seasons.

**Table 1:** Effect of different treatments on the gaseous exchange parameters of coriander at different stages under drought stress.

Treatments	Growth stage	PAR(µm/m^2^/s)	*E*(mmol/m^2^/s)	*C*(mmol/m^2^/s)	Int. CO_2_(µmol/mol)	Pn(µm/m^2^/s)	*Ѱ*_w_(MPa)	RWC(%)
First season (2019–2020)								
TRT1	VS	997.72 ± 0.56a	3.53 ± 0.08c	526.65 ± 0.72a	546.8 ± 0.38e	36.4 ± 0.17a	-0.08 ± 0.04a	88.6 ± 0.23a
	BS	814.05 ± 1.14f	1.49 ± 0.16c	77.16 ± 0.91c	424.6 ± 0.91c	15.09 ± 0.24a	-0.20 ± 0.06b	85.3 ± 1.74b
	SFS	445.85 ± 0.76b	1.82 ± 0.12ab	71.92 ± 4.12c	646.2 ± 0.97b	15.30 ± 0.19b	-0.54 ± 0.03b	75.1 ± 0.39b
TRT2	VS	998.42 ± 0.61a	3.62 ± 0.13c	526.9 ± 0.49a	546.7 ± 0.33e	36.48 ± 0.12a	-0.06 ± 0.06a	89.4 ± 1.09a
	BS	819.12 ± 0.9e	2.14 ± 0.16b	98.29 ± 1.37b	484.4 ± 1.08b	14.00 ± 1.33a	-0.13 ± 0.05a	90.9 ± 0.30a
	SFS	456.55 ± 0.99a	2.28 ± 0.15a	93.73 ± 2.87b	546.8 ± 0.82c	16.28 ± 0.33a	-0.42 ± 0.08a	81.7 ± 0.78a
TRT3	VS	933.72 ± 1.05c	4.60 ± 0.30a	421.35 ± 1.61b	647.6 ± 0.59b	22.36 ± 0.34c	-0.19 ± 0.02c	81.7 ± 0.48c
	BS	828.72 ± 0.47d	1.50 ± 0.14c	75.16 ± 0.88c	416.0 ± 0.71d	8.45 ± 0.29b	-0.27 ± 0.14c	74.5 ± 0.71c
	SFS	419.65 ± 1.34d	0.99 ± 0.30cd	65.93 ± 3.33c	523.3 ± 1.49e	6.73 ± 0.22d	-0.67 ± 0.11d	61.6 ± 0.47d
TRT4	VS	954.61 ± 0.73b	4.49 ± 0.22ab	369.99 ± 0.79c	579.5 ± 0.57d	28.84 ± 0.47b	-0.15 ± 0.01b	84.5 ± 0.65b
	BS	873.54 ± 1.24a	4.59 ± 0.12a	456.11 ± 1.13a	585.2 ± 0.74a	13.94 ± 0.80a	-0.20 ± 0.05b	86.4 ± 0.65b
	SFS	423.87 ± 0.84c	1.37 ± 0.29bc	122.58 ± 3.49a	534.7 ± 1.03d	7.55 ± 0.17c	-0.58 ± 0.15c	67.4 ± 0.84c
TRT5	VS	857.27 ± 0.62e	4.5 ± 0.24ab	348.68 ± 0.66d	619.0 ± 0.11c	18.52 ± 0.83d	-0.42 ± 0.02e	61.6 ± 0.79e
	BS	833.07 ± 1.20c	1.53 ± 0.25c	65.66 ± 1.08e	392.3 ± 0.84e	3.92 ± 0.21d	-0.53 ± 0.03e	56.5 ± 0.71e
	SFS	400.25 ± 1.65e	0.65 ± 0.05d	41.35 ± 3.30d	349.6 ± 0.67f	5.82 ± 1.19e	-0.76 ± 0.07e	49.9 ± 0.46f
TRT6	VS	916.65 ± 0.76d	3.94 ± 0.41bc	234.60 ± 0.80e	734.0 ± 0.92a	18.48 ± 0.39d	-0.32 ± 0.12d	66.4 ± 1.04d
	BS	842.55 ± 1.71b	1.53 ± 0.19c	69.31 ± 0.30d	484.7 ± 0.99b	6.17 ± 0.27c	-0.47 ± 0.16d	66.0 ± 0.50d
	SFS	379.75 ± 0.36f	1.46 ± 0.20bc	63.17 ± 3.23c	752.6 ± 0.36a	6.74 ± 0.25d	-0.67 ± 0.1d	54.3 ± 0.44e
Second season (2020–2021)								
TRT1	VS	979.97 ± 0.31a	3.47 ± 0.18b	527 ± 0.39a	539.9 ± 0.40f	36.55 ± 0.17a	-0.06 ± 0.03a	90.0 ± 0.36a
	BS	808 ± 0.88e	1.38 ± 0.15c	71.32 ± 0.36c	396.2 ± 0.64d	14.28 ± 0.19b	-0.23 ± 0.02b	86.8 ± 1.48b
	SFS	392.75 ± 1.67b	1.92 ± 0.26b	63.30 ± 0.50d	574.7 ± 0.21b	13.20 ± 0.49b	-0.55 ± 0.04b	74.1 ± 0.68b
TRT2	VS	976.42 ± 0.59b	3.43 ± 0.13b	527.35 ± 0.41a	544.9 ± 0.74e	35.74 ± 0.12a	-0.04 ± 0.08a	89.6 ± 0.71a
	BS	810.05 ± 1.25d	2.24 ± 0.23b	102.50 ± 0.32b	447.1 ± 0.41c	16.12 ± 0.64a	-0.16 ± 0.05a	90.8 ± 0.17a
	SFS	407.25 ± 0.41a	2.42 ± 0.21a	90.34 ± 0.38b	491.3 ± 0.56c	15.21 ± 0.58a	-0.49 ± 0.08a	79.6 ± 0.94a
TRT3	VS	883.05 ± 0.94d	4.41 ± 0.38a	431.81 ± 0.67b	607.7 ± 0.30b	25.12 ± 0.34c	-0.18 ± 0.16c	81.7 ± 0.60c
	BS	819.53 ± 0.47c	1.63 ± 0.17c	66.18 ± 0.36d	350.0 ± 0.62e	8.95 ± 0.49d	-0.30 ± 0.18c	73.4 ± 0.54d
	SFS	357.72 ± 0.91c	1.21 ± 0.26c	52.01 ± 0.80e	473.4 ± 0.34d	6.60 ± 0.34d	-0.71 ± 0.04d	60.2 ± 0.45d
TRT4	VS	866.82 ± 0.97e	4.49 ± 0.17a	370.07 ± 0.77c	577.3 ± 0.36d	27.15 ± 0.47b	-0.12 ± 0.13b	84.8 ± 0.58b
	BS	831.95 ± 0.73a	4.54 ± 0.23a	255.34 ± 0.45a	590.8 ± 0.66a	11.96 ± 0.37c	-0.25 ± 0.15b	83.7 ± 0.71c
	SFS	359.56 ± 1.37c	1.52 ± 0.07bc	119.84 ± 0.67a	448.5 ± 1.25e	9.22 ± 0.14c	-0.63 ± 0.14c	69.4 ± 0.72c
TRT5	VS	787.85 ± 0.17f	4.1 ± 0.50ab	338.14 ± 0.32d	588.8 ± 0.60c	17.20 ± 0.83e	-0.48 ± 0.11e	61.7 ± 0.66e
	BS	822.13 ± 0.99b	1.44 ± 0.21c	61.11 ± 0.26f	331.8 ± 0.45f	4.49 ± 0.35f	-0.56 ± 0.07e	55.6 ± 0.46e
	SFS	331.72 ± 0.93d	0.64 ± 0.04d	38.67 ± 0.38f	299.3 ± 0.20f	6.40 ± 0.37d	-0.79 ± 0.12e	48.7 ± 0.62f
TRT6	VS	886.72 ± 0.69c	3.93 ± 0.28ab	215.62 ± 0.47e	692.6 ± 0.35a	20.37 ± 0.39d	-0.37 ± 0.06d	66.8 ± 1.16d
	BS	823.21 ± 0.85b	1.49 ± 0.19c	63.35 ± 0.60e	454.7 ± 0.92b	7.88 ± 0.19e	-0.51 ± 0.05d	63.4 ± 0.46f
	SFS	329.25 ± 0.73d	1.61 ± 0.13bc	75.69 ± 0.49c	693.2 ± 1.04a	6.75 ± 0.45d	-0.69 ± 0.05d	55.6 ± 0.38e

The values are the mean ± SE of four replicates. Values with different letters indicate a significant difference between samples by least significant difference (p ≤ 0.05).

TRT, treatment; TRT1, US100; TRT2, US100 + folic acid (50 mM); TRT3, MS75; TRT4, MS75 + folic acid (50 mM); TRT5, SS50; TRT6, SS50 + folic acid (50 mM); VS, vegetative stage; BS, bolting stage; SSF, seed filling stage; PAR, photosynthetic active radiation; E, transpiration rate; C, stomatal conductance; Int. CO_2_, internal carbon dioxide; Pn, net photosynthetic rate.

The transpiration rate (*E*) in US100 and MS75 is non-significant, while SS50 showed significant results in the first season, whereas in the second season US100 and SS50 showed significant results as shown in **Table 1**. The maximum rates of transpiration (4.60 and 4.49 mmol/m^2^/s) were found at the vegetative stage in TRT3 and TRT4 when treated with FA (50 mM) followed by TRT5 as indicated in **Table 1** in both the first and second seasons. The minimum rate of transpiration was observed in TRT1 and TRT2 in both seasons. However, the transpiration rate showed a non-significant effect in all treatments in the bolting stage except TRT4 plants. The maximum rate of transpiration (4.59 and 4.54 mmol/m^2^/s) was found in MS75 plants when treated with FA as indicated in TRT4 at the bolting stage in both the first and second seasons. The minimum rate of transpiration was observed in TRT1 plants (control), whereas the optimum rate of transpiration at the seed filling stage was found in US100 plants when treated with FA (50 mM) as indicated in TRT2 in both seasons. The lowest rate of transpiration was observed in SS50 untreated plants (TRT5).

The stomatal conductance (C) at the vegetative stage was found to be maximum in TRT1 and TRT2 plants in both seasons, followed by TRT3 and TRT4, as presented in **Table 1**. The minimum *C* was found in TRT6 when SS50 was treated with FA respectively. Significant results were found of *C* in MS75, US50, and US100 plants at the bolting stage. The optimum stomatal conductance (456.11 and 255.34 mmol/m^2^/s) was found when MS75 plants were treated with FA in both seasons, followed by the plants of US100, as presented in TRT4 in **Table 1**. The highest *C* was found in TRT4 plants, followed by TRT2 plants, at the seed filling stage.

The highest amounts of Int. CO_2_ was 734.0 and 692.6 µmol/mol were found when SS50 plants were treated with FA (50 mM), as indicated in TRT6 in **Table 1**, at the vegetative stage in the first and second seasons, while the lowest amount of Int. CO_2_ was found in US100 plants. When plant came across the bolting stage, the maximum value was found in TRT4 (585.2 and 590.8 µmol/mol) plants, followed by TRT6 plants, and the least value was found in TRT5. The amount of Int. CO_2_ in leaves was significantly different in US100 plants and drought stress regimes (MS75 and SS50) in both seasons at the seed filling stage. The highest average mean of Int. CO_2_ was found in SS50 plants compared with untreated FA plants, followed by US100 plants.

A considerable increase in Pn was observed at the vegetative stage in TRT1 (36.40 and 36.55 µm/m^2^/s) and TRT2 (treated with FA, 50 mM) plants in both seasons. Under drought stress, the maximum Pn was observed in MS75 plants as indicated in TRT4 in **Table 1** for both seasons when plants were treated with FA (50 mM). The minimum Pn was measured in SS50 plants (TRT5). At the onset of the bolting stage, significant results were found between US100 plants as well as under drought stress regimes (MS75 and SS50) related to Pn. US100 plants treated with FA (50 mM) showed a substantial improvement in Pn in two consecutive seasons, followed by moderate drought stress plants (MS75), as indicated in **Table 1**. The lowest Pn was calculated in untreated SS50 plants. Furthermore, under drought stress regime, MS75 plants treated with FA showed a remarkable Pn rate compared with SS50 plants. Hence, the optimum Pn was found in TRT2 at the seed filling stage.

The water potential (*Ѱ*_w_) of leaf at the vegetative stage significantly decreased in MS75 plants (-0.19 and -0.18 MPa) and SS50 plants (-0.42 and 0.48 MPa) in TRT3 and TRT5 compared with US100 plants (-0.07 and -0.05 MPa), as presented in **Table 1**, in the first and second seasons, while the foliar application of FA improved the *Ѱ*_w_ to 21 and 23% in both water deficit regimes under treatment TRT4 and TRT6 (MS75: -0.15 MPa and SS50: -0.32 MPa) in the first season compared with untreated FA plants at the vegetative stage. Similar results that enhanced the *Ѱ*_w_—33% in MS75 plants (-0.12 MPa) and 23% in SS50 plants (-0.37 MPa)— were obtained in the second season after the application of FA, whereas at the bolting stage, *Ѱ*_w_ was significantly decreased in MS75 plants (-0.27 and -0.30 MPa) and SS50 plants (-0.53 and -0.56 MPa) compared with the fully irrigated plants (-0.20 and -0.16 MPa) of the first and second seasons. Meanwhile, the foliar application of FA improved the *Ѱ*_w_ to 26% (MS75) and 11% (SS50) in both water deficit regimes (-0.20 and -0.47 MPa) in the first season compared with the untreated FA plants. Significant results were obtained after the application of FA that enhanced the *Ѱ*_w_—17% in MS75 plants (-0.25 MPa) and 9% in SS50 plants (-0.51 MPa)—in the second season compared with their respective control plants (-0.30 and -0.56 MPa). Similarly, FA improved the *Ѱ*_w_ to 12% (MS75) and 13% (SS50) in both water deficit regimes under treatment—TRT4 and TRT16 (-0.58 and -0.67 MPa)—in the first season (**Figure 4C**). compared with the untreated FA plants (TRT3 and TRT5) at the seed filling stage. Significant results were obtained after the application of FA that enhanced the *Ѱ*_w_—11% in MS75 plants (-0.63 MPa) and 13% in SS50 plants (-0.69 MPa)—in the second season (**Table 1**) compared with the control plants (-0.71 and -0.79 MPa). Overall, foliar-applied FA showed a significant improvement in the *Ѱ*_w_ at different growth stages.

The results presented in **Table 1** indicate that RWC decreased significantly at the vegetative stage under drought stress. This decrease was 8 and 9% in MS75 plants and 31% in SS50 plants compared with US100 plants in two consecutive seasons. Remarkably, the plants treated with FA (50 mM) increased the RWC of the leaves of coriander in 3 and 8% in MS75 and 1R50 in both seasons, although RWC decreased significantly at the bolting stage under drought stress. The reduction in RWC was 13% in MS75 and 34% in IR50 in contrast to US100 plants during the bolting stage in the first season and 15 and 36% in the second season. The FA-treated plants showed an increment in RWC of the leaves of coriander in MS75 plants (16 and 14%) and 1R50 (17 and 14%) in both seasons compared with the untreated FA plants. Additionally, the well-watered plants also showed a significant increase of 7 and 5% in TRT2 compared with TRT1 plants in both consecutive seasons. At the seed filling stage, the decline in RWC was 18 and 19% in MS75 and 33 and 35% in SS50 compared with the control plants in the first and second seasons, respectively. The foliar application of FA enabled the coriander plants to retain water in the leaves of MS75 and SS50 in the first season (67 and 54%) and in the second season (69 and 56%) at the seed filling stage. The suggested results indicate that FA has the potential to improve the RWC under drought stress.

### Assessment of antioxidative enzymes

**Figure 7:**
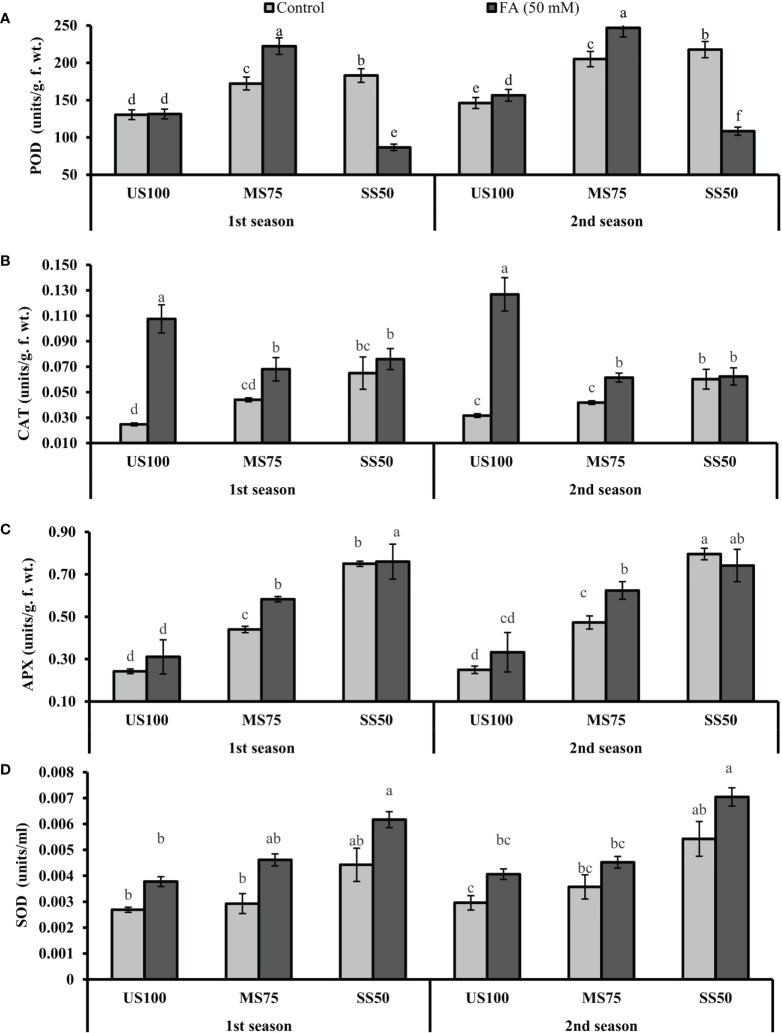
Effect of foliar application of folic acid (50 mM) on antioxidative enzymes **(A)** peroxidase, **(B)** catalase, **(C)** ascorbate peroxidase, and **(D)** superoxide dismutase in the first and second seasons. The graph values are the mean ± SE of four replicates. The bars exhibited with different letters indicate a significant difference between samples by least significant difference (*p* ≤ 0.05).

The presented results revealed that the activity of POD significantly increased to 32% (172.33 units/g. f. wt.) and 42% (180.00 units/g. f. wt.) in MS75 and SS50 plants compared with the well-watered plants (130.50 units/g. f. wt.) under drought stress in the first season (**Figure 7A**). Similar results that showed a substantial increase in POD activity to 40% (205.07 units/g. f. wt.) and 49% (217.77 units/g. f. wt.) in MS75 and SS50 plants compared with control plants (146.16 units/g. f. wt.) were obtained in the second season. Plants of MS75 treated with FA revealed that the activity of peroxidase enzyme increased to 29% (222.43 units/g. f. wt.) and 20% (246.89 units/g. f. wt.), while under intense drought stress (SS50), FA treatment decreased the activity of POD to 53% (86.75 units/g. f. wt.) and 50% (108.44 units/g. f. wt.) in the first and second seasons compared with the untreated FA plants (as indicated in **Figure 7A**).

The activity of CAT was likewise prominently enhanced to 78% and more than onefold in MS75 and SS50 plants in the first season, while the same activity at 32% and more than onefold was observed in the second season compared with the control plants. The foliar application of FA enhanced the activity of CAT to 55% (0.0680 units/g. f. wt.) and 17% (0.0760 units/g. f. wt.) in MS75 and SS50 plants in the first season. However, in the second season, the activity of CAT was increased to 47% (0.0613 units/g. f. wt.) in MS75 plants and 4% (0.0623 units/g. f. wt.) in SS50 plants compared with the control plants (**Figure 7B**).

However, the APX enzyme also showed a high activity under drought stress at nearly 81 and 74% in MS75 plants (0.440 and 0.473 units/g. f. wt.) and more than one-and-a-half-fold in SS50 plants of water deficit regimes compared with fully irrigated plants in both consecutive seasons. Furthermore, the results revealed that the FA-treated plants had an improved APX activity in MS75 plants (32%) plants in both seasons, non-significant results in SS50 plants were shown in the first season and a decreased activity of APX to 7% in the second season as presented in **Figure 7C**.

Similarly, under moderate drought stress, a marginal increase in the activity of SOD was found in MS75 plants. Nevertheless, a noticeable increment was found in severe drought stress (SS50). The maximum increase in SOD activity was 64 and 83% in SS50 plants, followed by 9 and 21% in MS75 plants under drought stress in both seasons. The activity of SOD was prominently enhanced in the FA-treated plants to 58 and 39% in MS75 and SS50 plants in the first season, while 27 and 30% improvement was observed in the second season compared with the US100 plants (**Figure 7D**). These results indicated that improvement in the activity of antioxidative enzymes under the influence of FA treatment alleviates the drought tolerance in coriander plants with respect to different drought regimes.

### Determination of Chla and Chlb content

**Figure 8:**
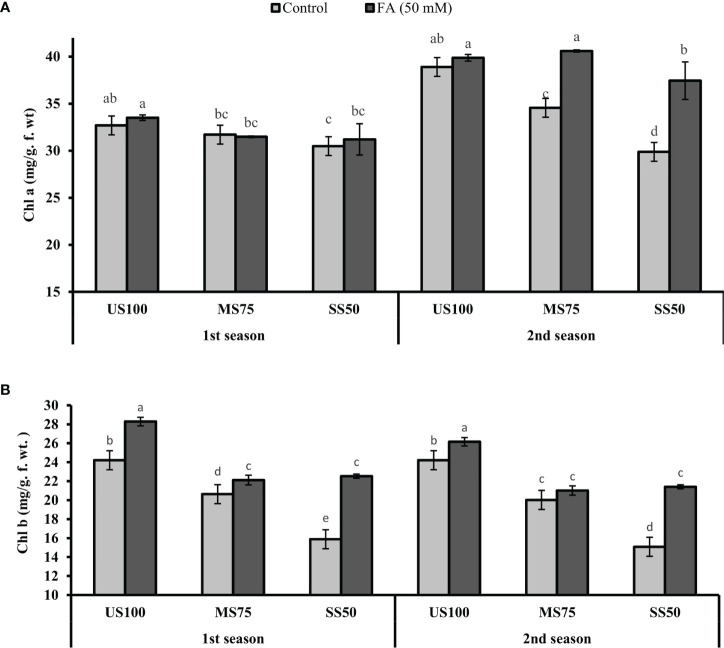
Effect of foliar application of folic acid (50 mM) on chlorophyll content **(A)** chlorophyll a and **(B)** chlorophyll b in the first and second seasons. The graph values are the mean ± SE of four replicates. The bars exhibited with different letters indicate a significant difference between samples by least significant difference (*p* ≤ 0.05).

The plants of MS75 showed a decrease of 3% (31.71 mg/g f. wt.) and 11% (34.56 mg/g f. wt.) in Chla content in the first and second seasons, respectively. However, the SS50 plants had a decreased Chla content to 7% (30.49 mg/g f. wt.) and 23% (29.88 mg/g f. wt.) in the first and second seasons compared with the US100 plants as indicated in **Figure 8A**. Similarly, Chlb also decreased under drought stress. In the first season, Chlb was reduced to 15% (20.63 mg/g f. wt.) in MS75 plants and 34% (15.88 mg/g f. wt.) in SS50 in comparison with the control plants. Furthermore, the results revealed that the FA-treated plants showed a non-significant effect on Chla content in MS75 plants in the first season, while in the second season FA improved the Chla content to 17% (40.60 mg/g f. wt.) in MS75 plants compared with the untreated plants. Furthermore, when the SS50 plants were treated with FA, the Chla content increased to 2% (31.20 mg/g f. wt.) and 25% (37.41 mg/g f. wt.) in the first and second seasons compared with the untreated FA plants as indicated in **Figure 8A**.

The plants of MS75, when treated with FA, showed that the content of Chlb improved to 7% (22.12 mg/g f. wt.) and 5% (21.01 mg/g f. wt.) in contrast to untreated FA plants in the first and second seasons, respectively. However, when the SS50 plants were treated with FA, Chlb significantly increased to 42% (22.53 mg/g f. wt.) and 38% (21.43 mg/g f. wt.) in the first and second seasons compared with the untreated FA plants as indicated in **Figure 8B**.

### Quantification of PRO and TSS

**Figure 9:**
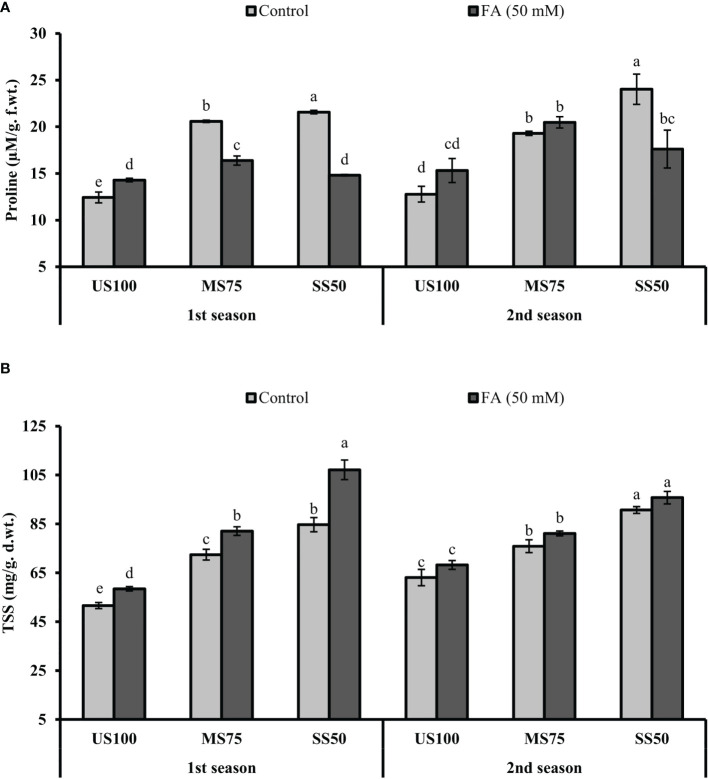
Effect of foliar application of folic acid (50 mM) on osmolytes **(A)** proline and **(B)** total soluble sugars in the first and second seasons. The graph values are the mean ± SE of four replicates. The bars exhibited with different letters indicate a significant difference between samples by least significant difference (*p* ≤ 0.05).

The presented results revealed that the concentration of PRO significantly increased to 66% (22.59 µM/g f.wt.) and 74% (21.57 µM/g f. wt.) in MS75 and SS50 plants compared with well-watered plants (12.43 µM/g f. wt.) under drought stress in the first season (**Figure 9A**). Similar results that showed a substantial increase in the concentration of PRO to 51% (19.30 µM/g f. wt.) and 88% (24.02 µM/g f.wt.) in MS75 and SS50 plants compared with control plants (12.78 µM/g f. wt.) were obtained in the second season, while the plants of MS75 treated with FA revealed that the concentration of PRO decreased to 20% (16.38 µM/g f. wt.) and showed a non-significant effect (20.48 µM/g f. wt.) in the first and second seasons, respectively. Nonetheless, when the SS50 plants were treated with FA, the concentration of PRO was decreased to 31% (14.81 µM/g f.wt.) and 27% (17.61 µM/g f. wt.) in the first and second seasons compared with the untreated FA plants as indicated in **Figure 9A**.

Further results revealed that the concentration of TSS significantly increased to 40% (72.42 mg/g d. wt.) and 64% (84.69 mg/g d. wt.) in MS75 and SS50 plants compared with the control plants (51.60 mg/g d. wt.) under drought stress in the first season (**Figure 9B**). Comparable results were obtained in the second season, which showed a substantial increase in the concentration of TSS to 20% (75.90 mg/g d. wt.) and 44% (90.71 mg/g d. wt.) in MS75 and SS50 plants compared with the control plants (63.05 mg/g d. wt.). However, the plants of MS75 treated with FA revealed that the concentration of TSS increased to 13% (82.07 mg/g d. wt.) and 7% (81.12 mg/g d. wt.) in the first and second seasons, respectively. Nonetheless, when SS50 plants were treated with FA, the concentration of TSS increased to 27% (107.34 mg/g d. wt.) and 6% (95.76 mg/g d. wt.) in the first and second seasons compared with the untreated FA plants as indicated in **Figure 9B**.

### Evaluation of TPC and TFC

**Figure 10:**
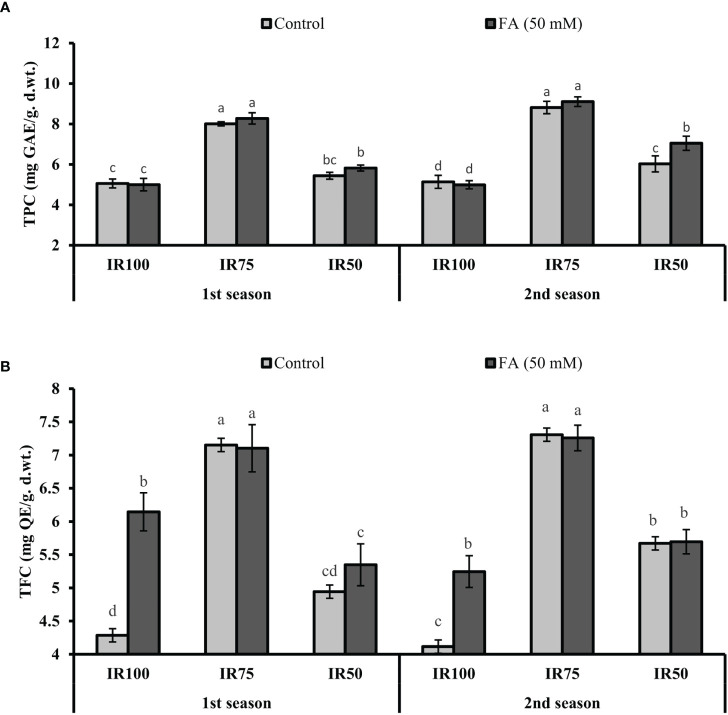
Effect of foliar application of folic acid (50 mM) on total phenolic content and total flavonoids content in the first season **(A)** and the second season **(B)** under different treatments. The graph values are the mean ± SE of four replicates. The bars exhibited with different letters show a significant difference by least significant difference (*p* ≤ 0.05).

Under drought stress regimes, TPC and TFC significantly increased to 58% (8.01 mg GAL Eq/g d. wt.) and 66% (7.15 mg QE/g d.wt.) in MS75 plants and 8% (5.44 mg GAL Eq/g d. wt.) and 15% (4.94 mg QC Eq/g d. wt.) in SS50 (TRT9) plants in the first season. A similar trend of significant increment in TPC and TFC was observed in MS75 plants (77 and 72%) and SS50 plants (17 and 37%) in the second season compared with fully irrigated US100 plants (**Figures 10A, B**).

The FA-treated plants significantly improved the TPC to 3% (8.28 mg GAL Eq/g d. wt.) and 3% (9.11 mg GAL Eq/g d. wt.) in MS75 plants, while 7% (5.82 mg GAL Eq/g d. wt.) and 17% (7.05 mg GAL Eq/g d.wt.) were observed in SS50 plants compared with the untreated FA plants in both consecutive seasons (**Figure 10A**). However, the TFC, because of the foliar application of FA in TRT4 (MS75), showed non-significant results compared with the untreated FA plants in both consecutive seasons. Furthermore, the plant of TRT6 (SS50) showed 8% (5.35 mg QC Eq/g d. wt.) increase in the first season and provided a non-significant result in the second year compared with the untreated FA plants under drought stress regimes (**Figure 10B**).

### Effects of different treatments on yield attributes

**Figure 11:**
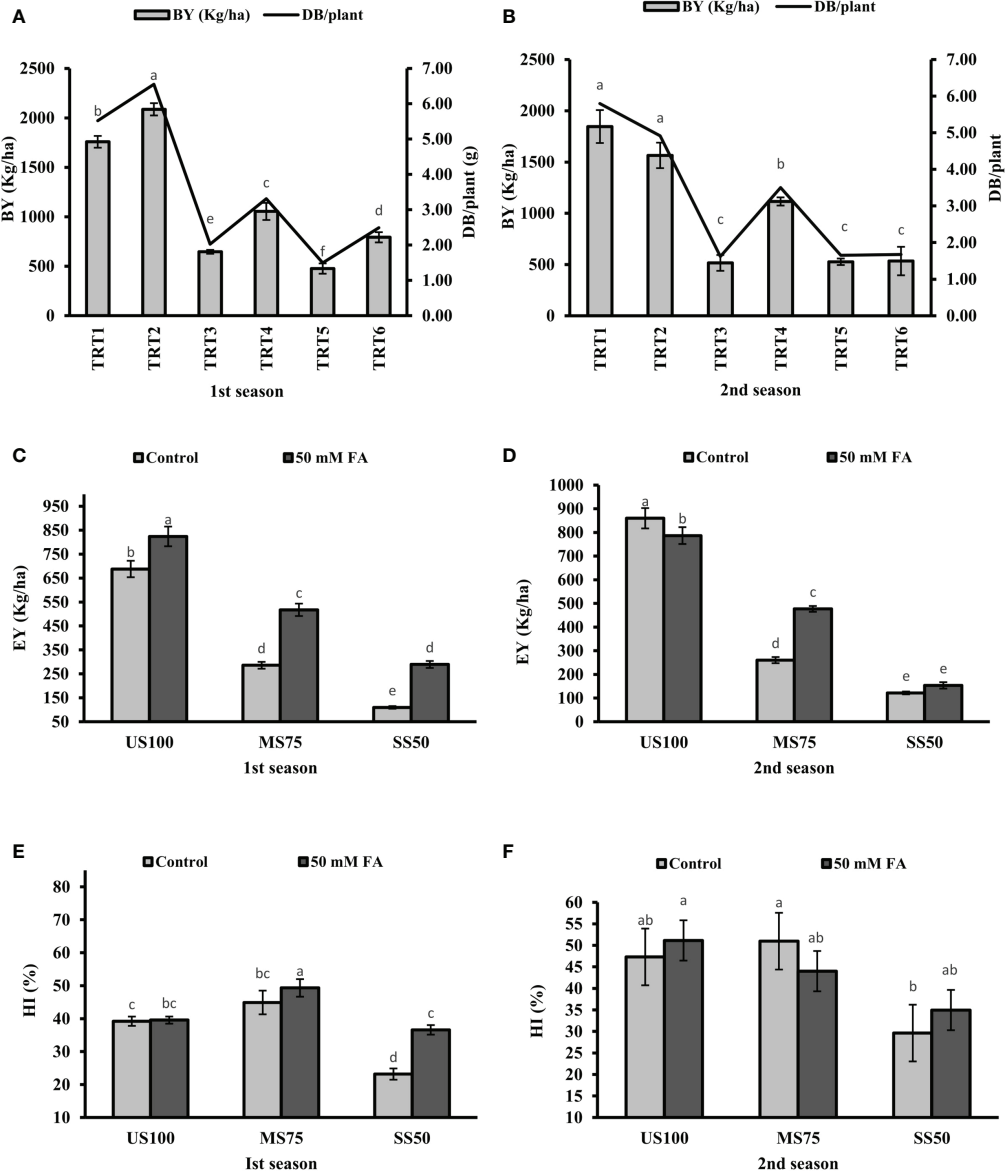
Effect of foliar application of folic acid (50 mM) on biological yield, economic yield, and harvest index in the first **(A, C, E)** and second **(B, D, F)** seasons. The graph values are the mean ± SE of four replicates. The bars exhibited with different letters indicate a significant difference between samples by least significant difference (*p* ≤ 0.05).

Under drought stress, BY significantly reduced to 63 and 72% in MS75 plants (TRT3) and 73 and 71% in SS50 (TRT6) plants in the first season and the second season compared with the US100 plants (**Figures 11A, B**). The foliar application of FA significantly increased BY to 63% (1,054 kg/ha) and more than onefold (1,115 kg/ha) in MS75 plants (both consecutive seasons), while an increment of 66% (792 kg/ha) in SS50 plants was observed in the first season and showed non-significant results (534 kg/ha) in the second season compared with the untreated FA plants.

Drought stress showed a significant effect and reduced the EY to 58% (286 kg/ha) and 70% (260 kg/ha) in MS75 plants (TRT3), while the reduction in SS50 plants (TRT5) was 84% (110 kg/ha) and 86% (122 kg/ha) in the first season and the second season compared with US100 plants (**Figures 11C, D**). In addition, under drought stress, HI in MS75 was 45 and 23% in SS50 of the first season. However, the HI of the second season was 51 and 30% in MS75 and SS50 plants (**Figures 11E, F**).

Under drought stress, the foliar application of FA significantly increase EY to 81% (518 kg/ha) and 83% (477 kg/ha) in MS75 plants (TRT4) and more than onefold (289 kg/ha) and 26% (154 kg/ha) in SS50 (TRT6) plants compared with the untreated FA plants in both consecutive seasons (**Figures 11C, D**). Plants treated with the foliar application of FA showed HI of 49 and 44% in MS75 plants (TRT4) and 37 and 35% in SS50 (TRT6) plants in both consecutive seasons (**Figures 11E, F**).

### Examination of DSI and DTE under water deficit conditions

**Figure 12:**
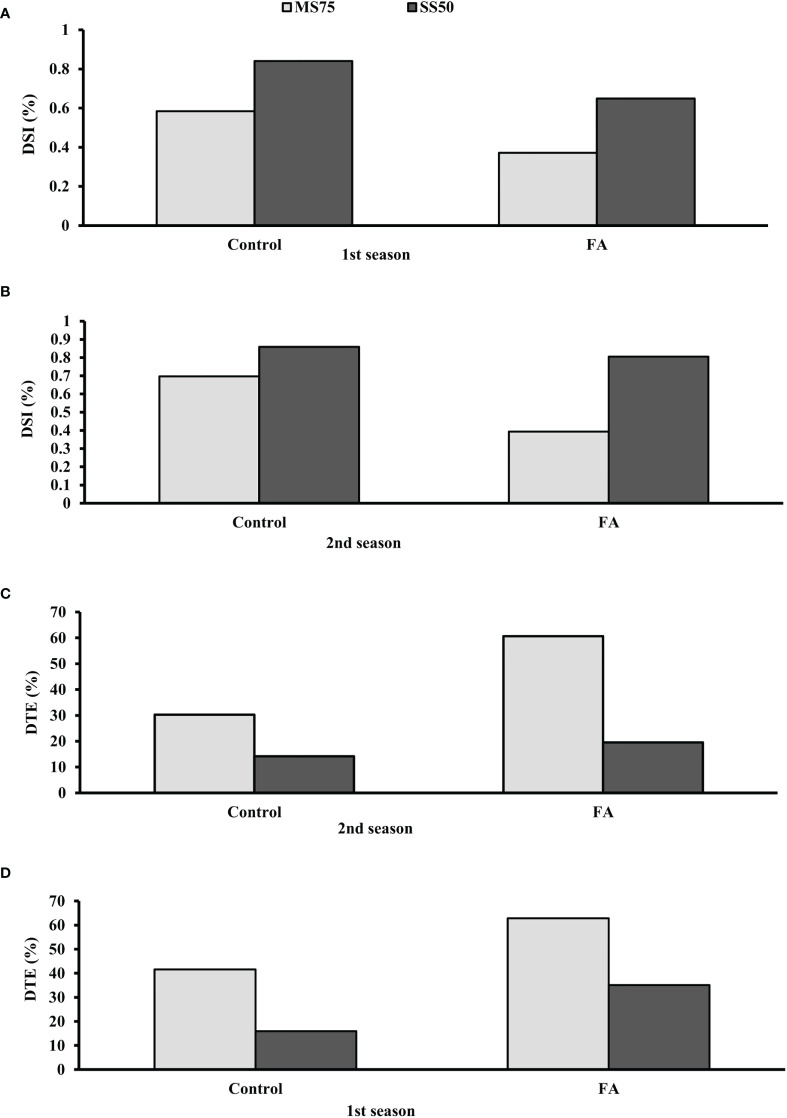
Effect of foliar application of folic acid (50 mM) on **(A, B)** drought susceptibility index and **(C, D)** drought tolerance efficiency in the first and second seasons.

The results demonstrated that plants of SS50 showed a high DSI compared with MS75 plants under the control conditions. The lowest DSI was found when SS50 plants were treated with FA. It was found that SS50 plants treated with FA (65 and 80%) showed a low DSI in both seasons compared with the control plants. Furthermore, the highest DSI (37 and 39%) was observed in MS75 plants in two consecutive seasons, respectively (**Figures 12A, B**). Hence, plants that faced severe drought stress (SS50) showed a high percentage of DSI compared with moderate stress (MS75) under the controlled environment, while FA-treated plants have the efficacy to ameliorate drought stress.

Data revealed that plants treated with FA showed better DTE in both stress regimes—MS75 as well as SS50 compared with unstressed plants. The greater DTE was found in MS75 plants when treated with FA (63 and 61%) in both seasons. Similarly, plants of SS50 treated with FA showed an improvement in DTE compared with the other treatments (**Figures 12C, D**).

### Production of EO% and OY under drought stress

**Figure 13:**
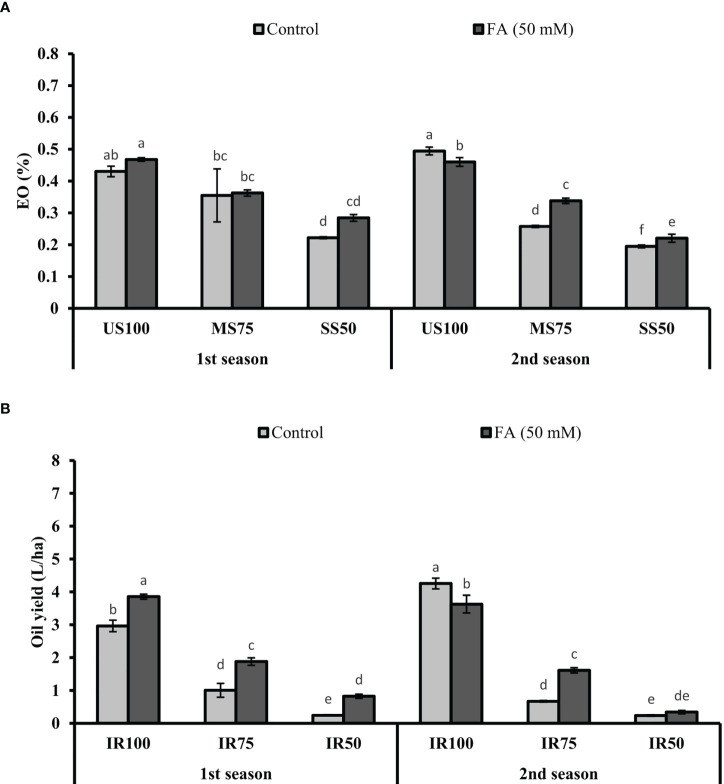
Effect of foliar application of folic acid (50 mM) on **(A)** % essential oil and **(B)** oil yield in the first and second seasons. The graph values are the mean ± SE of four replicates. The bars exhibited with different letters indicate a significant difference between samples by least significant difference (*p* ≤ 0.05).

The maximum EO% (47 and 43%) was observed in US100 plants when treated with FA, followed by untreated plants, in two consecutive seasons (**Figure 13A**). The EO% of MS75 and SS50 plants showed non-significant results in the first season and slightly significant results in the second season with 34 and 22% of EO.

The production of oil in terms of yield depicted that the maximum values, 3.86 and 3.63 L/ha, were obtained in US100 plants treated with FA (4 L/ha). As for MS75 plants, they showed enhanced OY of 1.88 and 1.61 L/ha compared with the untreated plants (1 and 0.67 L/ha) in both seasons (**Figure 13B**). Under severe drought stress (SS50), plants treated with FA showed a significant OY: more than three quarters L/ha and one quarter L/ha in the first and second seasons corresponding to the untreated plants.

### Upshot of treatments on the chemical constituents of EO through GC–MS analysis

The GC–MS data described the different constituents of EO with respect to their optical absorbance as mentioned in the “Materials and methods” section. The optimum abundance of linalool that was observed in all treatments indicates the major component of EO of coriander at RT (retention time). The GC–MS results revealed a higher abundance of linalool in all treatments regardless of water deficit conditions at RT 6.25. The maximum abundance of linalool (more than 60%) was found in MS75 and SS50 plants treated with FA under drought conditions in both seasons as indicated in **Table 2**. The second most abundant chemical component was α-terpeniol in the control plants as well as drought stress regimes at RT 21.32, followed by terpinene and p-cymene at RT 10.83 and 5.94, respectively. However, in US100 plants, α-terpeniol was relatively more abundant than in the water deficit regimes, although β-pinene was found to be the lowest chemical component found in EO.

**Table 2:** Relative (%) abundance of chemical constituents of EO detected through gas chromatography–mass spectrometry affected by different treatments under drought stress in the first and second seasons.

		First season						Second season					
RT	Compound	US100		MS75		SS50		US100		MS75		SS50	
		Control	FA	Control	FA	Control	FA	Control	FA	Control	FA	Control	FA
4.31	β- Pinene	0.12	0.11	0.12	0.14	0.14	0.09	0.16	0.06	0.18	0.19	1.28	0.26
5.94	P-Cymene	5.29	5.09	3.22	2.98	4.16	4.91	5.88	4.38	4.11	3.99	5.51	4.6
6.56	Linalool	62.39	63.41	64.04	66.76	60.01	61.31	63.38	63.4	64.31	63.42	62.87	66.11
9.43	Borneol	0.71	0.69	0.39	0.41	0.67	0.76	0.58	1.38	1.24	1.06	1.02	1.08
10.83	Terpinene	4.99	5.11	4.91	5.81	6.34	5.04	5.76	5.96	5.01	5.95	5.58	5.61
12.48	Nerol	2.55	3.18	3.12	3.41	6.21	5.2	3.96	4.1	4.12	4.31	5.02	5.97
14.85	Limonene	1.86	1.71	1.51	1.45	2.41	2.3	1.68	1.6	2.06	2.08	2.06	1.01
16.27	Geranyl acetate	1.02	1.48	1.42	1.21	0.89	0.79	0.71	0.82	0.76	0.81	1.39	0.41
21.32	α-Terpeniol	6.31	6.11	5.83	5.85	5.33	4.94	7.18	7.43	5.07	6.48	4.6	5.18
21.97	Geranyl acetate	1.97	3.02	1.55	1.59	1.61	1.5	1.78	3.61	1.44	1.21	0.68	0.7
23.47	Camphor	2.11	1.98	2.51	2.61	2.21	2.1	3.47	1.81	3.7	2.8	1.83	1.62
24.15	Undecanal	3.86	2.29	4.21	2.93	4.02	4.06	2.21	3.02	3.89	2.68	3.73	3.71
24.84	Thymol	0.81	0.62	1.8	0.99	1.67	1.8	0.64	0.26	1.01	0.99	1.58	2.02
25.12	Decanal	1.82	1.96	1.3	1.62	2.16	2.15	1.25	1.44	1.66	2.74	1.86	1.41
26.62	n-Octanal	0.72	0.16	0.37	0.41	0.09	0.01	0.88	0.39	0.52	0.67	0.06	0.23
27.56	Dillapiole	0.68	0.32	0.72	0.3	0.52	0.39	0.41	0.18	0.56	0.11	0.88	0.21
28.86	Geraniol	2.04	1.87	2.21	1.19	1.09	1.82	1.55	2.79	2.09	1.04	1.22	1.98
	Total	99.25	99.11	99.23	99.66	99.53	99.17	99.72	99.86	99.08	99.38	99.07	99.92

EO, essential oil; RT, retention time; US100, unstressed (full irrigation at field capacity); MS75, moderate stress at 75% field capacity of US100; SS50, severe stress at 50% field capacity of US100.

### Interpretation of PCA

Eigenvalues within the scree plots indicated three PCs that explained the 89.76 and 89.13% variation within the presented dataset of six treatments of the first and second seasons. The extracted eigenvector values showed the impact of PCs (biochemical and yield attributes) in drought tolerance within different treatments. The biplot results revealed that seven variables (Chla, Chlb, BY, EY, HI, EO, and OY) in PC1, five variables (POD, PRO, TPC, TFC, and HI) in PC2, and eight variables (CAT, SOD, Chla, Chlb, TSS, TPC, TFC, and HI) in PC3 showed significantly weak to moderate positive loadings out of the total of 15 variables for the determination of drought tolerance within six treatments; the rest of them showed negative loadings. Large positive loadings (EY, POD, and Chla) and large negative loadings (APX, SOD, and POD) determined the strong relationship and major contribution particularly to PC1, PC2, and PC3 in terms of drought tolerance in both consecutive seasons as shown in the supplementary material (**Supplementary Figure S1**). Thus, with reference to PC analysis, PC1 and PC2 exhibited 51 and 24% variation in the first season and 62 and 17% in the second season variation within the dataset of 15 variables. Hence, these two PCs described 75 and 79% of variance. Further results suggested that the foliar application of FA improves the stress tolerance in MS75 plants (TRT3 and TRT4, where both treatments fall in the positive part of the bi-plot) rather than in SS50 plants (TRT5 and TRT6, where both treatments fall in the negative part of the bi-plot) of PCA.

## Discussion

Global climate is the key cause of drought stress mainly due to water shortage as a result of altered rainfall patterns and distribution across the wide regions of the world (Mostofa et al., 2018). In addition to shortage of water input from rainfall, the loss of water from soil surface due to evaporation, further intensified by high temperature events, high light intensity, and dry wind, can aggravate an already existing drought stress condition (Ilyas et al., 2021). Thus, drought stress exhibits a prolonged multifaceted nature and has an adverse impact on the morphological, biochemical, molecular, physiological, and photosynthetic properties of herbaceous plants like coriander (Kaur and Asthir, 2017). As a result, a study of the agronomic performance of selected coriander was done in order to find improvement in drought tolerance and yield under stress conditions. The effect of drought stress regimes on coriander development at different growth stages has not been extensively researched. Drought stress affects the coriander at several phases of development—particularly at the vegetative, bolting, and seed filling stages (Kadhim, 2021). The vegetative stage manages the overall phenotypic expression of coriander and prepares for the upcoming reproductive phase. However, in the case of this study, we found that the most sensitive stage of growth with respect to drought stress regimes (MS75 AND SS50) was the seed filling stage (Sachdeva et al., 2022). The amount of reduction of plant and leaf fresh and dry biomass was greater and significant (*p* ≤ 0.05) during the seed filling stage rather than the bolting and vegetative stages (Khatun et al., 2021) as indicated in **Figures 5**, **6**, while shoot fresh and dry biomass showed non-significant results. Our findings indicating that the reproductive stage is the most vulnerable to SS50 in yield production is consistent with previously reported results (Rani et al., 2020). When plants are first exposed to MS75 and SS50, reduction in aerial growth rate occurs due to the restricted supply of water that initially generates the ROS in coriander and subsequently affects the plant metabolic machinery through physiological and biochemical changes (Khan et al., 2021).

In lieu of the above-mentioned context, gaseous exchange features were also affected under drought stress regimes (MS75 and SS50) in coriander plants, particularly the stomatal conductance (C) that may decrease the other associated physiological traits at different growth stages (Yan et al., 2016). Our results showed that *C* decreased gradually from vegetative to bolting and bolting to seed filling stages. The earliest response of the plant to prevent from desiccation might be through stomatal closure under drought stress conditions (Hura et al., 2007). As water deficit conditions prolonged during the development of the plant, severe effects have been observed on gaseous exchange attributes like transpiration rate (*E*), net photosynthesis (Pn), and concentration of internal carbon dioxide (Int. CO_2_). Initially at the vegetative stage, the decline in *C* did not affect the E and Pn significantly, but as the plant attained the bolting stage, the effect of *C* considerably decreased the E and Pn, which continued to the seed filling stage. Furthermore, we found that the *C* value showed a greater response than those of E and Pn at different growth stages under different intensities of drought stress, indicating that the vital role of *C* in regulating E and P*n* is consistent with the findings of previous studies (Yan et al., 2016; Soto et al., 2022). However, there is a high extent of co-regulation that was observed between *C*, E, and Pn—the decrease in E and Pn was smaller compared with *C* due to osmotic adjustment on account of the FA application (Rouhi et al., 2007).

The RWC of coriander indicated a slight decrease in MS75, followed by a significant decrease under SS50, which is in harmony with the results of Galmes et al. (2007). RWC, being metabolically accessible in water, may specify the metabolic role in leaf tissues, and it decreases with mild (MS75) to severe drought (SS50) stress regimes. LWP, which could indicate water availability to leaves, similarly decreases with mild to severe drought stress; hence, both RWC and LWP could serve as drought stress indicators for coriander. In this study, we discovered that the RWC values were lower than the LWP, indicating that LWP was more sensitive than RWC. This study supports LWP as an earlier indication of drought than RWC, which was in contradiction to the report of Sinclair and Ludlow’s assumption that RWC was a better option to assess drought tolerance.

Gas exchange is documented to be closely linked to leaf water scenario, which might be interpreted as a sign of stress under drought conditions (Jones, 2007). In the current study, we observed that gas exchange had a close association to leaf water attributes since prior research revealed that Pn in plants declined when RWC and LWP decreased (Lawlor, 2002; Yan et al., 2016; Soto et al., 2022).

All the above-mentioned quality parameters discussed are associated with each other for the operation of a metabolic machinery in plants. Therefore, a Pearson correlation was obtained for the evaluation of inter-relationship of growth and water and gaseous exchange features. Initially, the vegetative stage showed a strong to weak negative correlation of *E* with plant biomass and water attributes that could be the result of ambient temperature, air current movements, and relative humidity and PAR. A higher relative humidity lowers the E. However, all the quality parameters showed a weak to strong positive correlation from the bolting stage to the seed filling stage as indicated in **Supplementary Tables S2A, B, 3A, B**, which demonstrated an overall improvement in water status, gaseous exchange features, and biomass of the plant.

Reports have demonstrated that the modulation of antioxidant activity of plants takes place by producing antioxidant enzymes under water deficit regimes to restrict the damage of cellular integrity (Laxa et al., 2019). In addition, drought conditions may lead to oxidative stress primarily produced due to free radicals such as superoxide anions and hydroxyl radical and non-radical molecules singlet oxygen and hydrogen peroxide as mentioned by Gorelova et al. (2017). Therefore, to mitigate the effects of oxidative stress triggered by drought stress, plants have developed an antioxidant system that contains antioxidant enzymes like APX, CAT, POD, and SOD which scavenge ROS (Seleiman et al., 2021). **Figure 7** demonstrates that MS75 and SS50 significantly enhanced the enzymatic activity of antioxidants (APX, CAT, POD, and SOD) compared with US100 plants. This increment in antioxidant activities could indicate the production of ROS and the development of a coping strategy to counteract the oxidative injury induced by drought stress (Kaur and Asthir, 2017). The production of SOD is the frontline defensive mechanism against ROS, as it is the unique antioxidant enzyme that dismutates superoxide anions and converts them to H_2_O_2_, later on scavenged by CAT and APX—thus, protecting plants from the negative impact of ROS (Emam and Helal, 2008; Cui et al., 2018). It is well documented that the accumulation of H_2_O_2_ can cause cytotoxic effects in plant tissues. Furthermore, it triggered the programmed death of peroxisome and photosynthetic pigments like chloroplast (Smirnoff and Arnaud, 2019). As a result, a higher SOD activity increases the expression of other antioxidative enzymes such as POD, CAT, and APX under MS75 and SS5 (Boguszewska et al., 2010; Belay et al., 2021; El-Yazied et al., 2022; Sheikhalipour et al., 2022). POD, on the other hand, accelerates the dehydration of phenolic and endolic compounds along with the diminished properties of H_2_O_2_ (Pandey et al., 2010). Furthermore, plants treated with 50 mM FA demonstrated a considerable increase in H_2_O_2_ scavenging ability, as shown by a significant improvement in APX and SOD activity under MS75 and SS50 (**Figure 7**), thus resulting in the reduction of H_2_O_2_ concentration. Therefore, in the current study, the foliar treatment of FA can alleviate the oxidative stress caused by drought stress, significantly improving the status of antioxidant enzymes as reported by Khan et al. (2021). A significant increase was observed in APX, SOD, and POD activity in coriander plants treated with FA as indicated in the study of Badawi et al. (2004) and Al-Elwany et al. (2022). It could be the constructive role of FA in the production of NADPH and transformation of homocysteine to methionine that is involved in the detoxification of ROS production (Gorelova et al., 2017). These findings endorsed the key role of FA in regulating the antioxidant enzyme activity and ROS scavenging under drought conditions.

Water deficit conditions suppress the photosynthetic rate by limiting the pigments like Chla and Chlb. A number of studies reported the reduction in chlorophyll content and photosynthetic activity under abiotic stress (Rasheed et al., 2020; Diaz-Valencia et al., 2021; Mehak et al., 2021), although this study revealed that Chla had non-significant results, while Chlb showed a significant decline under MS75 and SS55 during both seasons (**Figure 8**). The reduction in Chlb might be attributed to the generation of ROS and activity of Chlb-degrading enzymes (Reddy and Vora, 1986; Taiz and Zeiger, 2010; Semida et al., 2021). Furthermore, the foliar nourishment of FA which improved the content of Chlb in coriander could be the result of the induction of the synthesis of glycine that is directly linked with the development of chromophores and chlorophyll as mentioned by Al-Maliky et al. (2019). It is well established that FA has a key role in the production of Chl content, which may coherently enhance the chlorophyll content in *C. sativum* under drought stress regimes (Gorelova et al., 2017). This finding was reinforced by the reports of Stakhova et al. (2000); Van Wilder et al. (2009); Kilic and Aca (2016), and Youssif and Youssif (2017) who found coherent results in pea, barley, and potato plants.

The properties possessed by osmolytes play a dynamic role towards drought tolerance as evidenced by the outcomes of the current study (**Figure 9**). The data presented showed that the PRO and TSS increased at high regimes of drought as in the cases of MS75 and SS50. Under water deficit conditions, both osmolytes (PRO and TSS) act as respiratory substrates to alleviate stress tolerance (Yooyongwech et al., 2016; Melaouhi et al., 2021). It could be the neutral nature of osmolytes that accumulates to prevent the denaturation of protein and cell membrane in the cytosol during drought conditions for osmotic adjustments and conservation of turgor pressure as described by Hebbache et al. (2021). The primary reason for the accumulation of proline may be the degradation of protein or the restriction of proline conversion under the action of drought stress (Sánchez et al., 1998). To counteract the prevalent condition of drought, PRO acts as ROS scavenger—maintaining the membrane integrity and structure of proteins and performing the function of molecular chaperones for osmotic balance to prevent cells from desiccation as reported in a number of studies (Parida et al., 2007; Ayub et al., 2021; Ibrahim et al., 2021; Singh et al., 2021; Rahimi et al., 2022). Similarly, TSS ameliorates drought tolerance through the process of osmoregulation and regulation of membrane integrity (El Sabagh et al., 2019). The foliar application of 50 mM FA significantly increased the content of TSS in coriander, while PRO showed a non-significant effect except at the moderate stress regime (**Figure 9**), which are in harmony with the findings of Rahimi et al. (2022). The possible mechanism behind the increment of osmolytes could be the involvement of FA in the biosynthesis of α-ketoglutaric acid, which reacts with ammonia to produce amino acids for protein synthesis and the production of plant hormones (IAA, CK, GAs, SA, and JA). It promotes plant metabolism, enzyme accumulation, cell division, and the production of osmolytes (Ibrahim et al., 2021; Khan et al., 2021).

With relevance to the abovementioned facts, the results of the present study demonstrated the increased production of bioactive compounds like TPC and TFC under MS75 and SS50 due to water deficit conditions. These are the secondary metabolites that assist the plant to maintain the osmotic balance and thus cope with water stress scenarios (Mashkor and Muhson, 2014; Bibi et al., 2022; Rahimi et al., 2022). Hence, the foliar supplementation of FA presented non-significant results of TPC and TFC at all levels of irrigation regimes (**Figure 10**). These results are contradictory to the findings of Dawood (2017). This could be due to the earliest mode of action of FA to activate the antioxidant system of coriander against drought stress to scavenge ROS rather than to promote the production of secondary metabolites (TPC and TFC) for water shortage conditions (Taghizadeh and Valizadeh Kaji, 2020).

Agricultural production in terms of BY and EY is devastatingly decreased due to drought stress as presented in **Figure 11**. The EY and BY of coriander significantly decline under the action of drought stress regimes. This reduction could be the consequence of modification in the functions of metabolic machinery. It includes reduction in leaf biomass, reduction in chlorophyll content, decline in photosynthetic rate, distorted gene expression under water stress, and limited distribution of photo-assimilates during seed filling that affects the overall EY and BYof coriander under MS75 and SS50 (Sah et al., 2020). Furthermore, the results revealed that HI was significantly reduced in SS50 rather than US100 and MS75 (**Figure 11**). It could be due to the effect of extreme water shortage on the different development stages that adversely decreased the overall yield (Sun et al., 2020). The increment in BY and EY may be attributed to the overall performance of FA under drought conditions during the growth of coriander metabolically, biochemically, and morphologically. Furthermore, HI was significantly reduced in severe water stress (SS50) rather than well or moderate water stress. It could be due to the effect of water shortage on the different development stages that adversely decreased the overall yield (Sun et al., 2020). These findings are inconsistent with previous reports about the significantly improved yield attributes of potato and sunflower when the water deficit regimes were treated with 50 mM FA (Ibrahim et al., 2015; Poudineh et al., 2015).

DSI is one of the scales that represent a decline in yield under drought stress treatment compared with the mean reduction of all the treatments and varieties (Fischer and Maurer, 1978). Moreover, this parameter confirms the drought tolerance of plants (Batieno et al., 2016). The presented results in **Figures 12A, B** revealed that the FA-treated plants showed a lower DSI compared with the untreated FA plants (control). Therefore, FA (50 mM) has the efficacy to ameliorate the drought tolerance in both MS75 and SS50 stress regimes. These suggested results followed the findings of Zdravković et al. (2013) and Golestani and Pakniyat (2007). Similarly, DTE is one of the other scales that measure the capability of plants to sustain the production of biological mass on the account of drought stress (Senthilkumar et al., 2021). Hence, FA-treated plants showed a significant improvement in DTE compared with the control due to better performance during the growth of coriander under drought conditions (**Figures 12C, D**).

It was well established that EO% and OY were remarkably increased under drought stress due to the accumulation of secondary metabolites (Afshari et al., 2021). However, the current study presented that well-watered plants revealed a higher EO% that further boosted up when plants were treated with FA as described by Unlukara et al. (2016) in **Figure 13A**. Overall, the EO% showed a non-significant effect of the different treatments in the present study except that a marginal improvement was observed under drought stress regimes when the plants of MS75 and SS50 were treated with FA. The results presented in **Figure 13B** revealed the valuable role of FA in the improvement of OY with respect to quality and quantity. These findings are in line with a previous study of Al-Elwany et al. (2022). The improvement in OY could be due to betterment in the water status of FA-nourished plants, leading to appropriate nutrient uptake, in response of which are the enhanced EO and OY contents (Rahimi et al., 2022). The chemical components of EO in all treatments showed a significant production of linalool in the drought regimes as well as in well-watered plants from 60 to 66% (**Table 2**), specifically in the case of FA treatment. This increment was according to the range described by Kaya et al. (2000). In addition, the other chemical constituents of EO, like limonene, α-pinene, terpinolene, p-cymene, nerol, thymol, camphor, undecanal, geranyl acetate, terpineol, and geraniol, did not cause any substantial change in the composition of EO under drought stress in both consecutive seasons (Pirbalouti et al., 2017). This may be due to the impact of FA in the modulation of the metabolic machinery of plants by increasing the concentration of secondary metabolites that are held responsible for the chemical composition of EO in drought stress regimes (Heydarnejadiyan et al., 2020; Mirzaie et al., 2020).

## Conclusion

The data presented in the results clearly showed that the foliar application of FA reduces the inhibitory impact of drought stress regimes in vegetative, bolting, and seed filling stages, thereby improving the growth traits (fresh and dry biomass), water attributes (*Ψ*_w_ and RWC), and gaseous exchange parameters. We found an increment in the antioxidant activity of enzymes (POD, SOD, and APX) and improvement in chlorophyll content (Chlb), osmolytes (TSS), and TFC that protect the metabolic machinery and cell integrity of coriander due to water deficit conditions. FA potentially improved BY, EY, and HI in moderate to severe stress plants compared with US100 plants. Furthermore, FA improved OY from 60 to 66% in two consecutive season experiments. The treatment of 50 mM substantially recovered the chemical constituents of EO, with an optimum concentration of linaloon followed by limonene, α-pinene, terpinolene, p-cymene, nerol, thymol, camphor, undecanal, geranyl acetate, terpineol, and geraniol. Generally, the findings indicated that foliar application of stress FA might ameliorate the negative impact of water deficit conditions on *C. sativum* development. Therefore, our study suggest that foliar supplementation of 50 mM FA can alleviate the harmful effects of water stress, which could be an appropriate method for reducing the harmful impact of water scarcity, mainly in arid regions where water scarcity is the core impediment to plant growth and performance. Furthermore, for a future perspective, we envision an important role of FA supplementation in drought stress tolerance in plants and thus push forward a new level of understanding of FA to cope with abiotic stress.

## Data availability statement

The original contributions presented in the study are included in the article/**Supplementary Material**. Further inquiries can be directed to the corresponding authors.

## Author contributions

MK designed the research idea; execute the experiment, data analysis, manuscript write up. SA supervised the research experiment, write and review the manuscript. RS and AA writing, editing and formal analysis. MS conceptualized the experiment and revised the manuscript. MM analyzed the data and assisted in editing and formatting. KA, NK, and SF provided with technical expertise to strengthen the basic idea of the research. SE, RM, and KG helped in revision, proofreading and funding acquisition for this publication. All authors helped in the revision of the article. All authors contributed to the article and approved the submitted version.

## Acknowledgments

The authors extend their appreciation to the Researchers Supporting Project number (RSP-2021/369), King Saud University, Riyadh, Saudi Arabia. Moreover, author’s pay special thanks to the lab fellows and technical staff of AEBRL for assisting in handling equipment’s and statistical software.

## Conflict of interest

The authors declare that the research was conducted in the absence of any commercial or financial relationships that could be construed as a potential conflict of interest.

## Publisher’s note

All claims expressed in this article are solely those of the authors and do not necessarily represent those of their affiliated organizations, or those of the publisher, the editors and the reviewers. Any product that may be evaluated in this article, or claim that may be made by its manufacturer, is not guaranteed or endorsed by the publisher.
