# Supplementary material for: Impression of foliar-applied folic acid on coriander (Coriandrum sativum L.) to regulate aerial growth, biochemical activity, and essential oil profiling under drought stress
Source: Front Plant Sci. 2022 Oct 21;13:1005710. doi: 10.3389/fpls.2022.1005710 (PMC9633984; doi:10.3389/fpls.2022.1005710)
Supplement: Supplementary file 1 [file DataSheet_1.docx]

Supplementary Material

Supplementary Table 1(A). Relationship between growth and water attributes versus gaseous exchange parameters of *C. sativum* at vegetative stage under drought stress in 1^st^ season

|  | PFB | LFB | SFB | Ψw | RWC | PAR | E | *C* | Int. CO2 | P*n* |
| --- | --- | --- | --- | --- | --- | --- | --- | --- | --- | --- |
| PFB | 1 |  |  |  |  |  |  |  |  |  |
| LFB | 0.95 | 1.00 |  |  |  |  |  |  |  |  |
| SFB | 0.89 | 0.72 | 1.00 |  |  |  |  |  |  |  |
| Ψw | 0.73 | 0.85 | 0.42 | 1.00 |  |  |  |  |  |  |
| RWC | 0.66 | 0.78 | 0.33 | 0.99 | 1.00 |  |  |  |  |  |
| PAR | 0.82 | 0.93 | 0.50 | 0.97 | 0.94 | 1.00 |  |  |  |  |
| E | -0.90 | -0.89 | -0.75 | -0.52 | -0.42 | -0.69 | 1.00 |  |  |  |
| *C* | 0.71 | 0.72 | 0.51 | 0.80 | 0.81 | 0.74 | -0.48 | 1.00 |  |  |
| Int. CO2 | -0.57 | -0.60 | -0.46 | -0.68 | -0.72 | -0.61 | 0.33 | -0.87 | 1.00 |  |
| P*n* | 0.85 | 0.92 | 0.61 | 0.91 | 0.90 | 0.92 | -0.68 | 0.87 | -0.85 | 1.00 |

PFB; Plant fresh biomass, LFB; Leaf fresh biomass, SFB; Shoot fresh biomass, Ψw; Water potential, PAR; Photosynthetic active radiation, E; Transpiration rate, C; Stomatal conductance, Int. CO_2_; Internal carbon dioxide, Pn; Net photosynthetic rate.

Supplementary Table 1(B). Relationship between growth and water attributes versus gaseous exchange parameters of *C. sativum* at vegetative stage under drought stress in 2^nd^ season

|  | PFB | LFB | SFB | Ψw | RWC | PAR | E | *C* | Int. CO2 | P*n* |
| --- | --- | --- | --- | --- | --- | --- | --- | --- | --- | --- |
| PFB | 1.00 |  |  |  |  |  |  |  |  |  |
| LFB | 0.99 | 1.00 |  |  |  |  |  |  |  |  |
| SFB | 0.82 | 0.76 | 1.00 |  |  |  |  |  |  |  |
| Ψw | 0.73 | 0.70 | 0.71 | 1.00 |  |  |  |  |  |  |
| RWC | 0.70 | 0.68 | 0.66 | 1.00 | 1.00 |  |  |  |  |  |
| PAR | 0.89 | 0.88 | 0.76 | 0.84 | 0.82 | 1.00 |  |  |  |  |
| E | -0.82 | -0.86 | -0.44 | -0.34 | -0.32 | -0.73 | 1.00 |  |  |  |
| *C* | 0.70 | 0.73 | 0.40 | 0.79 | 0.83 | 0.68 | -0.50 | 1.00 |  |  |
| Int. CO2 | -0.54 | -0.59 | -0.19 | -0.64 | -0.68 | -0.42 | 0.37 | -0.90 | 1.00 |  |
| P*n* | 0.88 | 0.88 | 0.66 | 0.93 | 0.92 | 0.92 | -0.65 | 0.86 | -0.73 | 1.00 |

PFB; Plant fresh biomass, LFB; Leaf fresh biomass, SFB; Shoot fresh biomass, Ψw; Water potential, PAR; Photosynthetic active radiation, E; Transpiration rate, C; Stomatal conductance, Int. CO_2_; Internal carbon dioxide, Pn; Net photosynthetic rate.

Supplementary Table 2(A). Relationship between growth and water attributes versus gaseous exchange parameters of *C. sativum* at bolting stage under drought stress in 1^st^ season

|  | PFB | LFB | SFB | Ψw | RWC | PAR | E | *C* | Int. CO2 | P*n* |
| --- | --- | --- | --- | --- | --- | --- | --- | --- | --- | --- |
| PFB | 1.00 |  |  |  |  |  |  |  |  |  |
| LFB | 0.95 | 1.00 |  |  |  |  |  |  |  |  |
| SFB | 0.20 | -0.04 | 1.00 |  |  |  |  |  |  |  |
| Ψw | 0.93 | 0.83 | 0.25 | 1.00 |  |  |  |  |  |  |
| RWC | 0.97 | 0.88 | 0.32 | 0.98 | 1.00 |  |  |  |  |  |
| PAR | -0.17 | -0.36 | 0.81 | -0.11 | -0.04 | 1.00 |  |  |  |  |
| E | 0.35 | 0.11 | 0.79 | 0.41 | 0.47 | 0.83 | 1.00 |  |  |  |
| C | 0.29 | 0.03 | 0.85 | 0.36 | 0.42 | 0.86 | 0.99 | 1.00 |  |  |
| Int. CO2 | 0.44 | 0.31 | 0.78 | 0.41 | 0.53 | 0.77 | 0.89 | 0.86 | 1.00 |  |
| P*n* | 0.86 | 0.93 | -0.27 | 0.72 | 0.75 | -0.60 | -0.10 | -0.17 | 0.01 | 1.00 |

PFB; Plant fresh biomass, LFB; Leaf fresh biomass, SFB; Shoot fresh biomass, Ψw; Water potential, PAR; Photosynthetic active radiation, E; Transpiration rate, C; Stomatal conductance, Int. CO_2_; Internal carbon dioxide, Pn; Net photosynthetic rate.

Supplementary Table 2(B). Relationship between growth and water attributes versus gaseous exchange parameters of *C. sativum* at bolting stage under drought stress in 2^nd^ season

|  | PFB | LFB | | SFB | Ψw | RWC | PAR | E | *C* | Int. CO2 | P*n* |
| --- | --- | --- | --- | --- | --- | --- | --- | --- | --- | --- | --- |
| PFB | 1 | |  |  |  |  |  |  |  |  |  |
| LFB | 1.00 | | 1.00 |  |  |  |  |  |  |  |  |
| SFB | 0.23 | | 0.23 | 1.00 |  |  |  |  |  |  |  |
| Ψw | 0.92 | | 0.92 | 0.33 | 1.00 |  |  |  |  |  |  |
| RWC | 0.97 | | 0.97 | 0.39 | 0.98 | 1.00 |  |  |  |  |  |
| PAR | -0.59 | | -0.58 | 0.53 | -0.44 | -0.45 | 1.00 |  |  |  |  |
| E | 0.28 | | 0.29 | 0.85 | 0.39 | 0.42 | 0.59 | 1.00 |  |  |  |
| *C* | 0.30 | | 0.31 | 0.88 | 0.39 | 0.43 | 0.56 | 0.99 | 1.00 |  |  |
| Int. CO2 | 0.38 | | 0.37 | 0.89 | 0.37 | 0.46 | 0.47 | 0.88 | 0.89 | 1.00 |  |
| P*n* | 0.96 | | 0.96 | 0.00 | 0.80 | 0.86 | -0.70 | 0.11 | 0.13 | 0.22 | 1.00 |

PFB; Plant fresh biomass, LFB; Leaf fresh biomass, SFB; Shoot fresh biomass, Ψw; Water potential, PAR; Photosynthetic active radiation, E; Transpiration rate, C; Stomatal conductance, Int. CO_2_; Internal carbon dioxide, Pn; Net photosynthetic rate.

Supplementary Table 3(A). Relationship between growth and water attributes versus gaseous exchange parameters of *C. sativum* at seed filling stage under drought stress in 1^st^ season

|  | PFB | LFB | SFB | Ψw | RWC | PAR | E | *C* | Int. CO2 | P*n* |
| --- | --- | --- | --- | --- | --- | --- | --- | --- | --- | --- |
| PFB | 1 |  |  |  |  |  |  |  |  |  |
| LFB | 1.00 | 1.00 |  |  |  |  |  |  |  |  |
| SFB | 0.14 | 0.16 | 1.00 |  |  |  |  |  |  |  |
| Ψw | 0.95 | 0.97 | 0.37 | 1.00 |  |  |  |  |  |  |
| RWC | 1.00 | 1.00 | 0.22 | 0.97 | 1.00 |  |  |  |  |  |
| PAR | 0.95 | 0.95 | -0.06 | 0.84 | 0.93 | 1.00 |  |  |  |  |
| E | 0.87 | 0.87 | 0.41 | 0.95 | 0.88 | 0.67 | 1.00 |  |  |  |
| *C* | 0.55 | 0.57 | 0.78 | 0.65 | 0.62 | 0.48 | 0.51 | 1.00 |  |  |
| Int. CO2 | 0.21 | 0.19 | 0.50 | 0.31 | 0.22 | -0.10 | 0.54 | 0.18 | 1.00 |  |
| P*n* | 0.91 | 0.90 | 0.39 | 0.87 | 0.93 | 0.85 | 0.76 | 0.77 | 0.28 | 1.00 |

PFB; Plant fresh biomass, LFB; Leaf fresh biomass, SFB; Shoot fresh biomass, Ψw; Water potential, PAR; Photosynthetic active radiation, E; Transpiration rate, C; Stomatal conductance, Int. CO_2_; Internal carbon dioxide, Pn; Net photosynthetic rate.

Supplementary Table 3(B). Relationship between growth and water attributes versus gaseous exchange parameters of *C. sativum* at seed filling stage under drought stress in 2^nd^ season

|  | PFB | LFB | SFB | Ψw | RWC | PAR | E | *C* | Int. CO2 | P*n* |
| --- | --- | --- | --- | --- | --- | --- | --- | --- | --- | --- |
| PFB | 1 |  |  |  |  |  |  |  |  |  |
| LFB | 1.00 | 1.00 |  |  |  |  |  |  |  |  |
| SFB | 0.52 | 0.55 | 1.00 |  |  |  |  |  |  |  |
| Ψw | 0.95 | 0.95 | 0.64 | 1.00 |  |  |  |  |  |  |
| RWC | 0.98 | 0.99 | 0.66 | 0.98 | 1.00 |  |  |  |  |  |
| PAR | 0.98 | 0.98 | 0.40 | 0.93 | 0.94 | 1.00 |  |  |  |  |
| E | 0.84 | 0.84 | 0.72 | 0.96 | 0.89 | 0.81 | 1.00 |  |  |  |
| *C* | 0.47 | 0.50 | 0.92 | 0.54 | 0.59 | 0.31 | 0.55 | 1.00 |  |  |
| Int. CO2 | 0.14 | 0.12 | 0.47 | 0.36 | 0.25 | 0.09 | 0.56 | 0.25 | 1.00 |  |
| P*n* | 0.98 | 0.98 | 0.66 | 0.99 | 1.00 | 0.94 | 0.92 | 0.58 | 0.32 | 1.00 |

PFB; Plant fresh biomass, LFB; Leaf fresh biomass, SFB; Shoot fresh biomass, Ψw; Water potential, PAR; Photosynthetic active radiation, E; Transpiration rate, C; Stomatal conductance, Int. CO_2_; Internal carbon dioxide, Pn; Net photosynthetic rate.


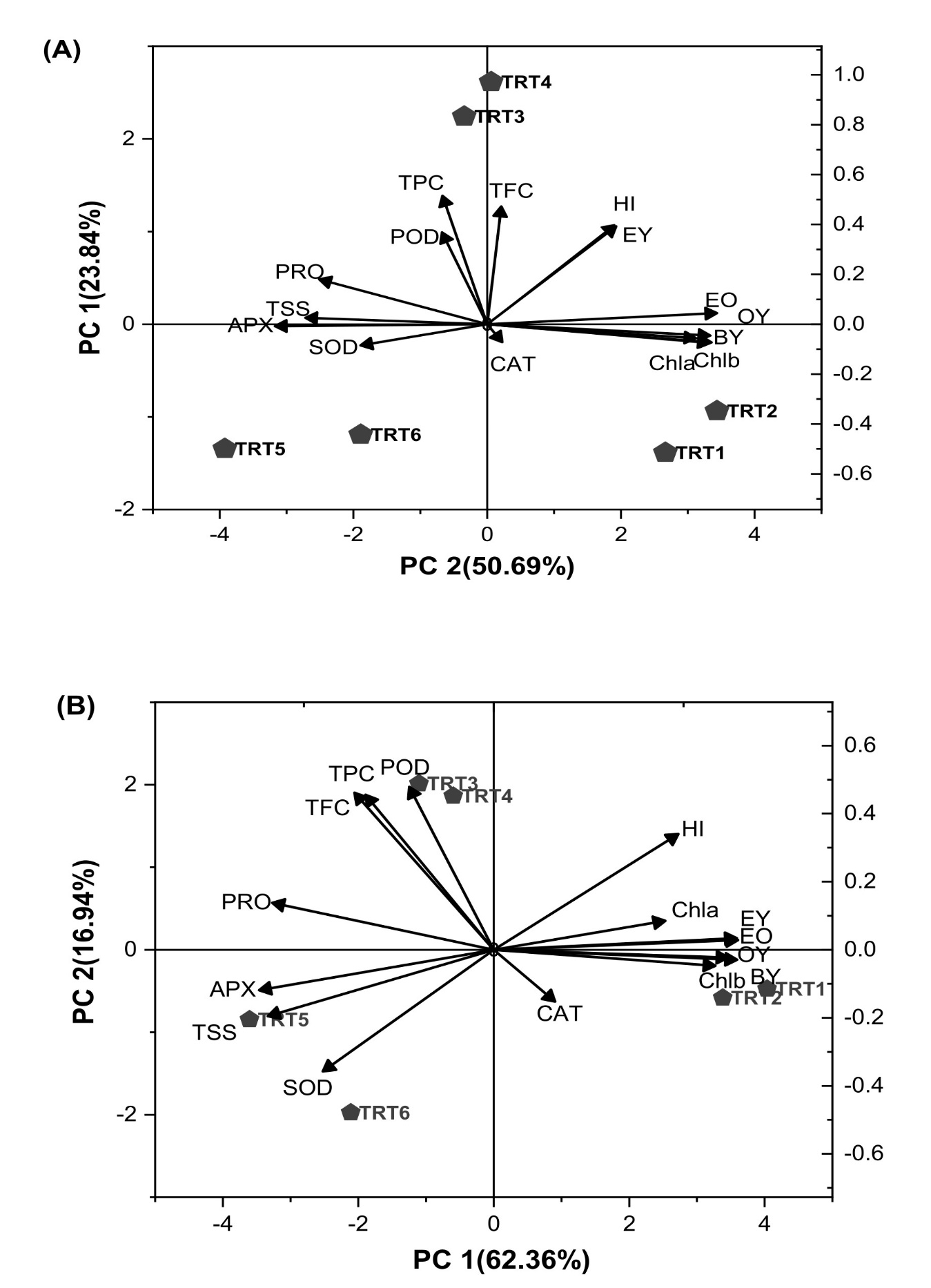


**Figure S1:** Principal component analysis (PCA) base on (A) 1^st^ season (B) 2^nd^ season
